# Cancer Screening Among Older Adults Above 75 Years of Age According to Health Status: A Population-Based Study

**DOI:** 10.1007/s11606-025-10037-3

**Published:** 2026-01-12

**Authors:** Frerik Smit, Axelle Braggion, Viktoria Gastens, Saman Khalatbari-Soltani, Stéphane Cullati, Arnaud Chiolero

**Affiliations:** 1 Population Health Laboratory (#PopHealthLab), University of Fribourg, Fribourg, Switzerland; 2 Swiss School of Public Health (SSPH+), Zurich, Switzerland; 3 School of Nursing Sciences, HES-SO University of Applied Sciences and Arts Western Switzerland, Lausanne, Switzerland; 4 Sydney School of Public Health, Faculty of Medicine and Health, University of Sydney, Sydney, NSW Australia; 5 Institute of Sociological Research, University of Geneva, Geneva, Switzerland; 6 Swiss Centre of Expertise in Life Course Research LIVES, Geneva, Switzerland; 7 Institute for Primary Health Care (BIHAM), University of Bern, Bern, Switzerland; 8 School of Population and Global Health, McGill University, Montreal, Canada

**Keywords:** Cancer screening, Older adults, Ervidence-based medicine, Public health

## Abstract

**Background:**

In the absence of evidence from trials, individualized risk-based approaches to cancer screening among older adults have been advocated to overcome the constraints of age-based screening recommendations. According to these risk-based approaches, older adults with poor health or multimorbidity should be less frequently or not screened given a lower likelihood of benefit due to lower life expectancy. We aimed to describe colorectal, breast, cervical, and prostate cancer screening among older adults above the age of 75 according to health status.

**Methods:**

Descriptive cross-sectional study analyzing the nationwide population-based 2022 Swiss Health Survey. Self-reported cancer screening in the past 12 months was assessed according to nine indicators of health status (self-rated health, chronic condition, number of morbidities, number of medications, activities of daily living, instrumental activities of daily living, functional limitations, smoking, and body mass index) with age- and sex-adjusted prevalence ratios (aPR) using modified Poisson regression.

**Results:**

A total of 2108 older adults were included (aged 76–80: 51%; women: 54%; tertiary education degree: 28%; multimorbidity (two or more morbidities): 49%; polypharmacy (five or more medications): 4%). The prevalence of any cancer screening in the past 12 months was 24.0% (men: 27.5%; women: 21.0%). These proportions were higher among older adults in poor health for five health status indicators but lower among those with less functional capabilities for the three corresponding indicators. Relative to older adults with no morbidity or medication use, any cancer screening was more common in those with multimorbidity (aPR for two–three morbidities: 1.40, 95% CI: 1.10–1.79; four or more morbidities: 1.66, 95% CI: 1.19–2.31) and polypharmacy (aPR: 1.54, 95% CI: 1.02–2.33).

**Conclusions:**

Cancer screening is common among older adults in poor health with multimorbidity and polypharmacy. Our findings reiterate the importance of expanding evidence on personalized approaches to cancer screening among older adults.

**Supplementary Information:**

The online version contains supplementary material available at 10.1007/s11606-025-10037-3.

## INTRODUCTION

Evidence on cancer screening effectiveness among older adults above 75 years of age is lacking due to this age group’s typical exclusion from trials.^1,2^ Accordingly, a majority of evidence-based guidelines across high-income countries generally stop recommending all forms of screening after the age of 75.^3–5^ This means that a significant proportion of cancer screening after the age of 75 can be considered a form of low-value care, which is care that does not bring a net benefit according to existing evidence.^6,7^ Nevertheless, screening above recommended ages is common, with an estimated four in ten older adults 75 years of age and above in Switzerland having been screened outside of recommendations.^8,9^ While not recommended, individualized risk-based approaches suggest that these screening practices could be beneficial if performed on older adults in good health with sufficient life expectancy.^1,2,10,11^ This is predicated on the notion of lag-time to benefit, whereby a time interval exists between when screening is undertaken and when a benefit can be observed.^11^ For breast and colorectal cancer screening, this interval has been approximated at ten years for averting one corresponding cancer-specific death per thousand screened individuals.^12^ In turn, it has been postulated that older adults whose life expectancy exceeds the lag-time to benefit of specific tests may benefit from screening irrespective of recommendations.^1,2,10,11^

In contrast, for older adults in poor health with multimorbidity or functional limitations — who thereby have a lower life expectancy^11,13^ — cancer screening is neither supported by evidence-based recommendations nor risk-based approaches. As such, older adults in poor health who screen for cancer are exposed to potential harm — including overdiagnosis^2^ — without evidence of a benefit. In this context, at the population level, older adults in better health should on average screen more than their less healthy counterparts. Correspondingly, to inform quality of care monitoring and efforts to de-implement low-value care,^14^ we aimed to describe overall and sex-stratified cancer screening among older adults above the age of 75 according to health status.

## METHODS

### Study Design and Data Source

This descriptive epidemiological study^15^ followed the Strengthening the Reporting of Observational Studies in Epidemiology (STROBE) checklist (Appendix Table 1).^16^ We analyzed the 2022 Swiss Health Survey (SHS), a nationwide cross-sectional population-based health survey utilizing state-stratified randomized probability sampling. A total of 60,651 residents of Switzerland 15 years of age and above were invited and 21,930 individuals participated (36.2% participation rate).^17^

### Study Population

Our target population was older adults above 75 years of age living in Switzerland. The study population comprised 2022 SHS participants aged above 75 (*n* = 2702). Our analytical sample included 2108 participants after excluding proxy respondents (*n* = 175) and participants with missing data on screening (*n* = 251) or the main health status indicators (*n* = 168) (Appendix Fig. 1, Appendix Table 2).

### Cancer Screening in Switzerland

Switzerland does not have an authoritative body that provides screening recommendations, yet certain organizations have produced recommendations specific to the Swiss context.^8^ This notably includes EviPrev, which recommends cervical and prostate cancer screening up to the age of 70, and colorectal and breast cancer screening up to the age of 75 — hence the selection of our target population of older adults above 75 years of age, as this is the age cut-off at which no screening is recommended for any cancer.^5^ EviPrev recommendations are explicitly informed by those from the United States Preventive Services Taskforce,^5^ and largely coincide with recommendations from other corresponding bodies internationally including the European commission.^3,4^ Certain cantons (states) in Switzerland have organized screening programs for breast or colorectal cancer, with upper age limits of 69 or 74 years for breast cancer screening and of 69 years for colorectal cancer screening.^18,19^ In most cantons, however, screening is opportunistic and offered through physicians.^18,19^

### Variables of Interest

Ascertainment details of included variables are reported in Appendix Tables 3–5 and elsewhere.^20^ Socio-demographic variables included age, sex, education level, and linguistic region.

#### Health Status

Indicators of health status were chosen based on availability within the SHS and their known association with life expectancy or inclusion within life expectancy estimators/indices for older adults (Appendix Table 6).^13,21–24^ Included variables were self-rated health (categorized into: good or very good, average, bad or very bad), having a chronic condition or long-term health issue (yes/no), number of morbidities (categorized into: zero, one, two–three, four or more), number of medications used (categorized into: zero, one–two, three–four, five or more), difficulties with slightly adapted activities of daily living (difficulty/no difficulty) and instrumental activities of daily living (difficulty/no difficulty) scales, functional limitations (difficulty/no difficulty), smoking status (categorized into: never smoker, former smoker, current smoker), and body weight category (categorized by body mass index (BMI) into: not obese (BMI < 30) and obese (BMI ≥ 30)). We created the indicator “number of morbidities” by combining participant responses on whether they had asthma, pulmonary disorders (bronchitis, chronic obstructive pulmonary disease, and emphysema), hypertension, elevated cholesterol, diabetes, myocardial infarction, stroke, cancer, and depression. Multimorbidity was defined as having two or more morbidities.^25^ The indicator “number of medications used” combined participants’ self-reported use of hypertension, heart, insomnia, pain, calming, attention, cholesterol, depression, diabetes, and osteoporosis medications. Polypharmacy was defined as using five or more medications.^26^

#### Cancer Screening

In line with the definition of screening as looking for cancer in individuals prior to any symptoms,^27^ we considered screening as detection tests used specifically for preventive screening purposes, and not as diagnostic tests. We assessed screening for colorectal (fecal occult blood test (FOBT) and colonoscopy), breast (mammography), cervical (cervical smear), and prostate (prostate specific antigen (PSA) test or rectal exam) cancer, as these are historically the most commonly recommended forms of population-based screening.^3^ SHS participants self-reported whether they had undergone these specific cancer tests along with the reason (preventive, diagnostic, or other) and timing of their last corresponding test. The outcome variables were colorectal, breast, cervical, and prostate cancer screening in the past 12 months. We created the variable “any cancer screening,” whereby participants who had undergone any of these screening tests were classified as having been screened. Similarly, we also created the variable “any colorectal cancer screening” for use of FOBT and/or colonoscopy.

### Statistical Analysis

We estimated the proportion of participants who were screened for cancer overall, by sex, by age, and according to health status indicators. Modified Poisson regression models with robust error variance were used to obtain prevalence ratios and 95% confidence intervals (CI) of cancer screening patterns according to health status.^28^ Separate models were run for each health status indicator, with both an unadjusted model and an age- and sex-adjusted model (age-adjusted for sex-stratified analysis). To provide an overall overview of cancer screening practices among older adults above 75 years of age, our main analysis focused on any cancer screening as the main outcome. We conducted additional analyses for each type of screening individually given variability in the value of screening across screening types.

The unadjusted prevalence ratios (PR) we reported show the actual pattern of cancer screening according to health status that occurred within our sample.^15,29^ The adjusted prevalence ratios (aPR) show the underlying screening pattern when our sample is imaginarily altered so that age and sex are balanced across health status groups.^29^ Of note, given that this is a descriptive study, this analytical adjustment was not intended to control for confounding.^15,30^ Rather, it was intended to remove differences between health status groups that, based on our judgment, are permissible explanations of potential differences in screening.^29^ More specifically, when comparing screening between older adults in good and poor health, any differences may partially be explained by these groups having different age and sex distributions. Correspondingly, as most cancer screening types are sex-specific^3^ and that both age- and life expectancy-based approaches to screening eventually recommend screening cessation after a certain age and remaining lifespan,^1–3,10,11,21,22,24^ screening differences between sexes and age groups can reasonably be deemed acceptable. Therefore, screening patterns according to health status that are merely reflective of potential age and sex differences in screening across health status groups are arguably less meaningful results for informing screening practices.

The Swiss Federal Office of Statistics provides survey weights designed to account for the SHS’s complex sampling design, adjust for non-response bias, and make results more nationally representative of the population of Switzerland 15 years of age and above through calibration. The variables used to calculate these weights included age, sex, region, civil status, household size and type, language, nationality, education, and revenue.^17^ Applying these weights should have transformed the study sample to better reflect the distribution of these variables according to their known distribution in the actual population of Switzerland 15 years of age and above.^31^ However, given that our study was restricted to older adults above 75 years of age, the transformed study sample produced by applying these weights might not accurately be representative of the distribution of the weighting variables in our target population (i.e., those above 75 years of age). Put differently, given that the weights were calibrated using a reference population that included, but extended beyond, our target population, they might have led to undesired weighting effects.^31^ Therefore, while we reported weighted results in our main analysis, we also reported unweighted results in the supplementary material.

### Sensitivity Analyses

We re-calculated results of the “any cancer screening” outcome for different constructions of the number of morbidities indicator, where hypertension and elevated cholesterol were excluded from the created variable (separately and together). This was because hypertension and elevated cholesterol are often screened for as risk factors of cardiovascular disease, thereby leading to their high prevalence in the Swiss population without necessarily being markers of poor health.^32^

## RESULTS

Of 2108 included participants, 51% were aged between 76 and 80, 54% were women, and 28% had a tertiary education degree. Most participants reported being in either good or very good health (72%) and having no difficulties with functional capabilities (Table 1).

**Table 1:** Characteristics of Included Participants (Weighted Proportions)

	All (***n*** = 2108)	Men (***n*** = 951)	Women (***n*** = 1157)
Age group			
76–80 81–85 86 +	50.8% 29.5% 19.7%	50.6% 30.0% 19.4%	50.9% 29.1% 20.0%
Sex			
Man Woman	46.2% 53.8%	100% -	- 100%
Education level			
Obligatory schooling Secondary degree Tertiary degree	17.1% 54.5% 27.5%	8.3% 47.7% 43.0%	24.7% 60.4% 14.1%
Linguistic region			
German French Italian	74.3% 21.3% 4.4%	75.9% 20.1% 4.0%	72.9% 22.3% 4.8%
Self-rated health			
Good or very good Average Bad or very bad	71.6% 24.0% 4.4%	76.0% 20.2% 3.8%	67.9% 27.2% 4.9%
Chronic condition or long-term health issue			
No Yes	45.8% 54.2%	47.3% 52.7%	44.4% 55.6%
Number of morbidities*			
0 1 2–3 4 or more	21.7% 29.2% 41.0% 8.1%	18.8% 27.3% 43.9% 10.0%	24.2% 30.8% 38.5% 6.5%
Number of medications**			
0 1–2 3–4 5 or more	22.0% 44.0% 29.5% 4.4%	21.5% 44.8% 30.1% 3.6%	22.5% 43.3% 29.1% 5.0%
Activities of daily living			
No difficulty Difficulty	91.4% 8.6%	92.0% 8.0%	91.0% 9.0%
Instrumental activities of daily living			
No difficulty Difficulty	59.1% 40.9%	67.4% 32.6%	52.0% 48.0%
Functional limitations			
No difficulty Difficulty	70.1% 29.9%	70.7% 29.3%	69.5% 30.5%
Smoking status			
Never smoker Former smoker Current smoker	54.7% 36.6% 8.7%	40.9% 49.3% 9.8%	66.5% 25.6% 7.9%
Body mass index			
Not obese Obese	86.8% 13.2%	87.3% 12.7%	86.3% 13.7%

^*^ Based on nine different morbidities: asthma, pulmonary disorders (bronchitis, COPD, and emphysema), hypertension, elevated cholesterol, diabetes, myocardial infarction, stroke, cancer, and depression

^**^ Based on 10 different medication types: hypertension medication, heart medication, insomnia medication, pain medication, calming medication, attention medication, cholesterol medication, depression medication, diabetes medication, and osteoporosis medication

Table 2 reports weighted proportions of cancer screening in the past 12 months across different screening types (Appendix Table 7 for unweighted). Overall, 24.0% of older adults above the age of 75 have been screened for any cancer (men: 27.5%; women: 21.0%). Screening for prostate (19.9% of men) and cervical cancer (14.4% of women) was the most common.

**Table 2:** Proportions of Cancer Screening in the Past 12 Months Among Older Adults Above 75 Years of Age (Weighted)

Cancer screening	All (*n* = 2108)	Men (*n* = 951)	Women (*n* = 1157)
Any cancer screening			
Yes No	24.0% 76.0%	27.5% 72.5%	21.0% 79.0%
Colorectal cancer screening (FOBT or colonoscopy)			
Yes No	9.4% 90.6%	12.4% 87.6%	6.7% 93.3%
Breast cancer screening (mammography)			
Yes No	- -	- -	5.5% 94.5%
Cervical cancer screening (cervical smear)			
Yes No	- -	- -	14.4% 85.6%
Prostate cancer screening (PSA or rectal exam)			
Yes No	- -	19.9% 80.1%	- -

*FOBT* fecal occult blood test, *PSA* prostate-specific antigen test

**Figure 1:**
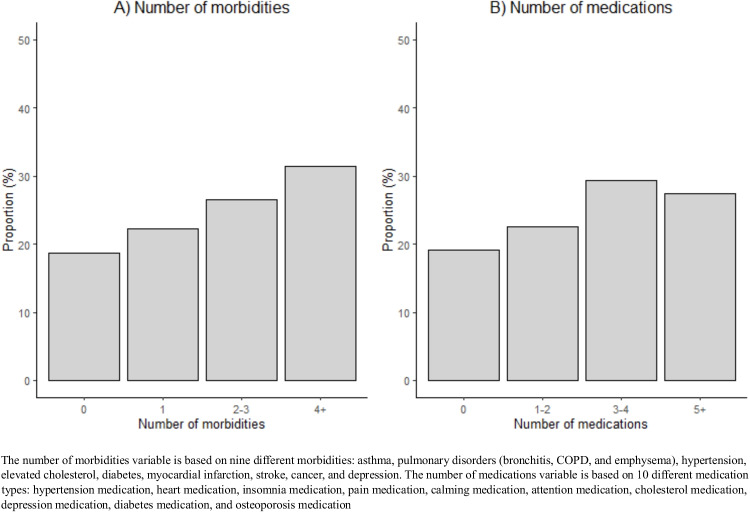
Proportions of any cancer screening in the past 12 months in categories of number of morbidities and medications among all older adults above 75 years of age (weighted).

Table 3 reports results for any cancer screening among all older adults (Appendix Table 8 for unweighted). Any cancer screening in the past 12 months decreased with increasing age (76–80: 30.2%; 81–85: 20.8%; 86 + 12.9%). Figure 1 depicts screening patterns according to the number of morbidities and medications, where screening was more common among older adults with multimorbidity (aPR for two–three morbidities: 1.40; 95% CI: 1.10–1.79; four or more morbidities: 1.66; 95% CI: 1.19–2.31) and polypharmacy (aPR for five or more medications: 1.54; 95% CI: 1.02–2.33) compared to those with no morbidities and medications, respectively. Screening was slightly higher among older adults with bad or very bad self-rated health compared to good or very good self-rated health, and among those with a chronic condition or a long-term health issue than those without. Obese older adults were screened more than not obese older adults. Patterns were mixed for smoking status, where former smokers were screened more than never smokers, but current smokers were screened less than never smokers. Finally, for all three indicators of functional capabilities, older adults with difficulties were screened less than those without.

**Table 3:** Proportions and Prevalence Ratios of Any Cancer Screening in the Past 12 Months in Categories of Health Status Indicators and Age Group Among All Older Adults Above 75 Years of Age (Weighted)

	All older adults (*n* = 2,108)		
Screening according to health indicators	Proportions	PR (unadjusted)	aPR (adjusted for age and sex)
Age group			
76–80 81–85 86+	30.2% 20.8% 12.9%	(ref) 0.69 (0.56–0.84) 0.43 (0.30–0.61)	- - -
Self-rated health			
Good or very good Average Bad or very bad	23.8% 24.4% 25.3%	(ref) 1.03 (0.83–1.27) 1.06 (0.69–1.63)	(ref) 1.08 (0.88–1.33) 1.15 (0.77–1.71)
Chronic condition or long-term health issue			
No Yes	22.2% 25.5%	(ref) 1.15 (0.96–1.37)	(ref) 1.17 (0.98–1.39)
Number of morbidities*			
0 1 2–3 4 or more	18.7% 22.2% 26.6% 31.5%	(ref) 1.19 (0.90–1.56) 1.42 (1.11–1.83) 1.68 (1.20–2.35)	(ref) 1.22 (0.93–1.59) 1.40 (1.10–1.79) 1.66 (1.19–2.31)
Number of medications**			
0 1–2 3–4 5 or more	19.1% 22.6% 29.3% 27.5%	(ref) 1.18 (0.91–1.54) 1.53 (1.17–2.00) 1.44 (0.95–2.17)	(ref) 1.19 (0.92–1.54) 1.57 (1.20–2.05) 1.54 (1.02–2.33)
Activities of daily living			
No difficulty Difficulty	24.5% 19.1%	(ref) 0.78 (0.55–1.11)	(ref) 0.93 (0.65–1.32)
Instrumental activities of daily living			
No difficulty Difficulty	27.1% 19.5%	(ref) 0.72 (0.60–0.87)	(ref) 0.85 (0.70–1.03)
Functional limitations			
No difficulty Difficulty	25.3% 21.0%	(ref) 0.83 (0.68–1.01)	(ref) 0.91 (0.75–1.12)
Smoking status			
Never smoker Former smoker Current smoker	22.5% 27.2% 19.9%	(ref) 1.21 (1.00–1.45) 0.88 (0.63–1.24)	(ref) 1.09 (0.90–1.32) 0.78 (0.56–1.08)
Body mass index			
Not obese Obese	23.5% 27.2%	(ref) 1.16 (0.90–1.48)	(ref) 1.07 (0.84–1.37)

*PR* prevalence ratio, *aPR* adjusted prevalence ratio

* based on nine different morbidities: asthma, pulmonary disorders (bronchitis, COPD, and emphysema), hypertension, elevated cholesterol, diabetes, myocardial infarction, stroke, cancer, and depression

** based on 10 different medication types: hypertension medication, heart medication, insomnia medication, pain medication, calming medication, attention medication, cholesterol medication, depression medication, diabetes medication, and osteoporosis medication

Weighted sex-stratified results are reported in Table 4 (Appendix Table 9 for unweighted) and indicate that screening patterns according to health status are generally similar across sexes, with certain sex differences for individual health indicators reflective of varying patterns across screening types (Appendix Tables 10–17). Notably, the pattern of more screening among older adults with multimorbidity and polypharmacy is present for both sexes.

**Table 4:** Proportions and Prevalence Ratios of Any Cancer Screening in the Past 12 Months in Categories of Health Status Indicators and Age Group Among Older Men and Women Above 75 Years of Age (Weighted)

	Men (*n* = 951)			Women (*n* = 1157)		
Screening according to health indicators	Proportions	PR (unadjusted)	aPR (adjusted for age and sex)	Proportions	PR (unadjusted)	aPR (adjusted for age and sex)
Age group						
76–80 81–85 86 +	32.8% 24.8% 18.2%	(ref) 0.76 (0.58–0.99) 0.56 (0.36–0.86)	- - -	28.0% 17.4% 8.4%	(ref) 0.62 (0.46–0.84) 0.30 (0.16–0.55)	- - -
Self-rated health						
Good or very good Average Bad or very bad	25.8% 34.1% 27.1%	(ref) 1.32 (1.01–1.73) 1.05 (0.56–1.97)	(ref) 1.28 (0.98–1.68) 1.08 (0.60–1.93)	21.9% 18.3% 24.1%	(ref) 0.83 (0.60–1.16) 1.10 (0.61–1.97)	(ref) 0.93 (0.67–1.29) 1.22 (0.71–2.10)
Chronic condition or long-term health issue						
No Yes	24.4% 30.4%	(ref) 1.25 (0.98–1.59)	(ref) 1.25 (0.98–1.58)	20.2% 21.6%	(ref) 1.07 (0.82–1.38)	(ref) 1.10 (0.85–1.42)
Number of morbidities*						
0 1 2–3 4 or more	21.7% 25.0% 29.9% 34.9%	(ref) 1.15 (0.77–1.73) 1.38 (0.96–1.98) 1.61 (1.03–2.52)	(ref) 1.20 (0.81–1.79) 1.36 (0.95–1.95) 1.59 (1.03–2.46)	16.7% 20.1% 23.4% 27.0%	(ref) 1.20 (0.83–1.74) 1.40 (0.98–1.99) 1.61 (0.94–2.76)	(ref) 1.23 (0.86–1.75) 1.46 (1.04–2.05) 1.82 (1.07–3.10)
Number of medications**						
0 1–2 3–4 5 or more	21.3% 25.4% 35.2% 27.5%	(ref) 1.19 (0.82–1.72) 1.65 (1.14–2.39) 1.29 (0.67–2.48)	(ref) 1.21 (0.84–1.74) 1.66 (1.15–2.40) 1.34 (0.70–2.53)	17.4% 20.1% 24.0% 27.5%	(ref) 1.16 (0.80–1.69) 1.38 (0.93–2.05) 1.58 (0.92–2.72)	(ref) 1.17 (0.81–1.69) 1.50 (1.02–2.22) 1.71 (1.00–2.94)
Activities of daily living						
No difficulty Difficulty	28.3% 19.1%	(ref) 0.68 (0.39–1.17)	(ref) 0.74 (0.43–1.28)	21.2% 19.0%	(ref) 0.90 (0.57–1.42)	(ref) 1.20 (0.77–1.88)
Instrumental activities of daily living						
No difficulty Difficulty	29.3% 23.9%	(ref) 0.82 (0.62–1.07)	(ref) 0.87 (0.67–1.14)	24.7% 17.0%	(ref) 0.69 (0.53–0.90)	(ref) 0.84 (0.64–1.12)
Functional limitations						
No difficulty Difficulty	28.7% 24.8%	(ref) 0.86 (0.66–1.14)	(ref) 0.92 (0.70–1.21)	22.4% 17.8%	(ref) 0.79 (0.59–1.07)	(ref) 0.91 (0.68–1.22)
Smoking status						
Never smoker Former smoker Current smoker	27.5% 27.1% 29.9%	(ref) 0.99 (0.77–1.27) 1.09 (0.74–1.61)	(ref) 0.98 (0.76–1.26) 1.00 (0.67–1.49)	19.9% 27.4% 9.3%	(ref) 1.37 (1.05–1.81) 0.47 (0.25–0.86)	(ref) 1.27 (0.96–1.66) 0.44 (0.24–0.80)
Body mass index						
Not obese Obese	26.4% 35.4%	(ref) 1.34 (0.99–1.82)	(ref) 1.25 (0.92–1.71)	21.0% 20.7%	(ref) 0.98 (0.64–1.50)	(ref) 0.90 (0.60–1.35)

*PR* prevalence ratio, *aPR* adjusted prevalence ratio

* Based on nine different morbidities: asthma, pulmonary disorders (bronchitis, COPD, and emphysema), hypertension, elevated cholesterol, diabetes, myocardial infarction, stroke, cancer, and depression

** Based on 10 different medication types: hypertension medication, heart medication, insomnia medication, pain medication, calming medication, attention medication, cholesterol medication, depression medication, diabetes medication, and osteoporosis medication

In the sensitivity analysis (Appendix Tables 18–21), removing hypertension and elevated cholesterol resulted in small strata for the four or more number of morbidities category. This is especially among women, leading to very few women in our sample being categorized as having four or more morbidities. Nevertheless, in all sensitivity analyses, older adults with two or more morbidities were screened more than those with zero morbidities, indicating that the pattern of more screening among older adults with multimorbidity generally remained.

## DISCUSSION

We described cancer screening among older adults above the age of 75 according to health status. Many people in this age group (all: 24.0%; men: 27.5%; women: 21.0%) were screened for cancer in the past 12 months despite it generally not being recommended.^3^ We also observed that screening proportions were higher among older adults in poor health for five health status indicators. Most notably, older adults with multimorbidity and polypharmacy were screened more. However, older adults with less functional capabilities were screened less.

Study limitations include the SHS’s 36.2% participation rate, whereby results may not be representative of our target population. More specifically, owing to healthy volunteer bias, people in poor health were likely underrepresented and people engaging in screening were likely overrepresented within our sample.^33,34^ This limited generalizability is further hampered by available survey weights not being calibrated directly to our target population, but rather a wider population inclusive of our target population. We therefore also calculated unweighted results, where weighted screening proportions were slightly lower than unweighted proportions, and among all older adults the prevalence ratios of cancer screening according to health status were similar irrespective of weighting. However, for certain results stratified by sex or screening type, some large weighting effects were observed in the highest categories of the number of morbidities and medications variables, which contained small strata.

Another limitation is that included health status indicators are unlikely to be fully indicative of a person’s overall health or predictive of their life expectancy, nor do they provide information as to the severity of morbidities. Accordingly, to capture health status as holistically as possible, we included a wide array of different types of health indicators covering subjective measures, disease and medications, and functional capabilities. BMI in particular is a complex health indicator among older adults and it remains unclear what the optimal BMI is at that age.^35^ We therefore report results with alternative BMI categorization in Appendix Tables 22–40. Data are also limited by being self-reported, which likely overestimated cancer screening.^36^ Insurance data has alternatively been used to analyze screening use in Switzerland, but they are limited in not differentiating between screening and diagnostic testing,^37^ which is a differentiation made in the SHS. Finally, our focus on screening in the past 12 months does not fully capture the prevalence of screening in our study population^8^ and may have impacted observed screening patterns if certain health status groups screen more regularly.

Our results add to existing literature on this topic, which has largely been limited to the United States context.^38^ Concordant with our findings, one review of breast cancer screening among older women found that an increased number of comorbidities was associated with more screening, while difficulties with activities of daily living and instrumental activities of daily living were associated with less screening.^39^ Similarly, a study on lung cancer screening among older adults also observed more screening among people with more comorbidities and less screening among people with more functional impairments.^40^ Correspondingly, the screening pattern we observed may be explained by healthcare utilization being greater among multimorbid individuals and people with polypharmacy,^41,42^ as well as individuals with multimorbidity being more inclined to demand low-value healthcare services.^43^ Meanwhile, our findings that older adults with functional difficulties were screened less may be the result of physical access barriers to engagement with preventive care services for those with reduced mobility.^44^

Regarding implications for healthcare practitioners and policymakers, our findings suggest that health status and life expectancy are likely not driving cancer screening decision-making for older adults. This is despite this framework being widely recommended, as screening individuals outside of recommendations with low life expectancy can be considered low-value likely resulting in unnecessary harm.^1,2,6,10,11^ While some practitioners already consider these factors,^45^ many have affirmed opposing views on what should drive screening decision-making.^46^ This coincides with an array of barriers to clinicians’ incorporation of life expectancy in cancer screening practice,^47^ including patient disagreement with screening according to life expectancy,^48^ and people holding views that drive their screening behaviors irrespective of recommendations.^49^ Correspondingly, it is important that cancer screening decision-making among older adults also takes into account the individual values and preferences of patients.^50^ To empower such patient-centered shared decision-making,^51^ there is a general need for improving cancer screening literacy within the population, particularly as patients and clinicians generally underestimate the harms of screening while overestimating its benefits.^52–54^

Critically, the potential effectiveness of risk-based approaches to cancer screening needs to be demonstrated in future research. Promisingly, certain trials evaluating risk-based approaches to cancer screening are currently being undertaken.^55,56^ However, such trials need to include older adults who have historically been excluded from screening trials.^1,2,57^ Additionally, trials should assess the lag-time to benefit of screening as an outcome.^11,12^ This is because screening according to life expectancy is contingent on having a screening threshold determined by the lag-time to benefit of the corresponding screening type.^11^ Yet, existing lag-time to benefit estimates have mainly been calculated through post-hoc analysis of survival curves in trial reports,^12^ which may be less reliable than primary outcome estimates. Similarly, life expectancy-based screening also requires accurate life expectancy estimators valid for their target populations. However, the generalizability of existing life expectancy estimators may be hindered due to selection bias, and they may not be transportable to external populations.^13^ This is particularly concerning from an equity perspective, as marginalized groups are typically underrepresented within health research.^58^ Any efforts to transition to risk-based screening must therefore take concerted efforts to ensure that such a transition does not exacerbate existing inequities in screening utilization and cancer outcomes.^58^

## CONCLUSION

Cancer screening among older adults above the age of 75 is common irrespective of health status despite generally not being recommended. For five health status indicators, screening was more common among older adults in worse health, including older adults with multimorbidity and polypharmacy. However, older adults with less functional capabilities screened less. Taken together, our findings reiterate the importance of expanding evidence on risk-based approaches to cancer screening, particularly among older adults.

## Supplementary Information

Below is the link to the electronic supplementary material.

ESM 1 (DOCX 162 KB)

## Data Availability

Data from the Swiss Health Survey 2022 are available for a fee (400 Swiss Francs, plus 7.7% tax) and users must request permission from the Swiss Federal Statistical Office (sgb@bfs.admin.ch). Data must be destroyed after 5 years.
