# Supplementary material for: Cancer Screening Among Older Adults Above 75 Years of Age According to Health Status: A Population-Based Study
Source: J Gen Intern Med. 2026 Jan 12;41(9):2498–506. doi: 10.1007/s11606-025-10037-3 (PMC13305071; doi:10.1007/s11606-025-10037-3)
Supplement: Supplementary file 1 — (DOCX 162 KB) [file 11606_2025_10037_MOESM1_ESM.docx]

**Supplementary Material**

**Study title:** Cancer screening among older adults above 75 years of age according to health status: a population-based study

**Table of Contents**

[Appendix Table 1 - Strengthening the reporting of observational studies in epidemiology (STROBE) checklist 7](#_Toc207095806)

[Appendix Figure 1 – Flowchart of analytical sample inclusion 10](#_Toc207095807)

[Appendix Table 2 – Missing data for each variable of interest among all non-proxy respondents of the Swiss Health Survey above 75 years of age 11](#_Toc207095808)

[Appendix Table 3 – Ascertainment of health status variables 12](#_Toc207095809)

[Appendix Table 4 – Ascertainment of cancer screening variables 17](#_Toc207095810)

[Appendix Table 5 – Ascertainment of socio-demographic variables 19](#_Toc207095811)

[Appendix Table 6 – Inclusion justifications for health status indicators 20](#_Toc207095812)

[Appendix Table 7 – Proportions of any cancer screening in the past 12 months among older adults above 75 years of age (unweighted) 21](#_Toc207095813)

[Appendix Table 8 – Proportions and prevalence ratios of any cancer screening in the past 12 months in categories of health status indicators and age group among all older adults above 75 years of age (unweighted) 22](#_Toc207095814)

[Appendix Table 9 – Proportions and prevalence ratios of any cancer screening in the past 12 months in categories of health status indicators and age group among older men and women above 75 years of age (unweighted) 23](#_Toc207095815)

[Appendix Table 10 – Proportions and prevalence ratios of any colorectal cancer screening in the past 12 months in categories of health status indicators and age group among all older adults above 75 years of age (weighted) 25](#_Toc207095816)

[Appendix Table 11 – Proportions and prevalence ratios of any colorectal cancer screening in the past 12 months in categories of health status indicators and age group among all older adults above 75 years of age (unweighted) 26](#_Toc207095817)

[Appendix Table 12 – Proportions and prevalence ratios of FOBT and colonoscopy screening in the past 12 months in categories of health status indicators and age group among all older adults above 75 years of age (weighted) 27](#_Toc207095818)

[Appendix Table 13 – Proportions and prevalence ratios of FOBT and colonoscopy screening in the past 12 months in categories of health status indicators and age group among all older adults above 75 years of age (unweighted) 29](#_Toc207095819)

[Appendix Table 14 – Proportions and prevalence ratios of breast and cervical cancer screening in the past 12 months in categories of health status indicators and age group among older women above 75 years of age (weighted) 31](#_Toc207095820)

[Appendix Table 15 – Proportions and prevalence ratios of breast and cervical cancer screening in the past 12 months in categories of health status indicators and age group among older women above 75 years of age (unweighted) 33](#_Toc207095821)

[Appendix Table 16 – Proportions and prevalence ratios of prostate cancer screening in the past 12 months in categories of health status indicators and age group among older men above 75 years of age (weighted) 35](#_Toc207095822)

[Appendix Table 17 – Proportions and prevalence ratios of prostate cancer screening in the past 12 months in categories of health status indicators and age group among older men above 75 years of age (unweighted) 36](#_Toc207095823)

[Appendix Table 18 - Sensitivity analysis: Proportions and prevalence ratios of any cancer screening in the past 12 months in categories of number of morbidities among all older adults above 75 years of age (weighted) 37](#_Toc207095824)

[Appendix Table 19 – Sensitivity analysis: Proportions and prevalence ratios of any cancer screening in the past 12 months according to number of morbidities among older men and women above 75 years of age (weighted) 38](#_Toc207095825)

[Appendix Table 20 - Sensitivity analysis: Proportions and prevalence ratios of any cancer screening in the past 12 months in categories of number of morbidities among all older adults above 75 years of age (unweighted) 39](#_Toc207095826)

[Appendix Table 21 – Sensitivity analysis: Proportions and prevalence ratios of any cancer screening in the past 12 months according to number of morbidities among older men and women above 75 years of age (unweighted prevalence ratios) 40](#_Toc207095827)

[Appendix Table 22 – Proportions of study participants in body mass index categories with alternative categorizations (weighted) 41](#_Toc207095828)

[Appendix Table 23 – Proportions of any cancer screening in the past 12 months in granular body mass index categories among older adults above 75 years of age (weighted) 42](#_Toc207095829)

[Appendix Table 24 – Proportions and prevalence ratios of any cancer screening in the past 12 months in body mass index categories with alternative categorization of body mass index among older adults above 75 years of age (weighted) 43](#_Toc207095830)

[Appendix Table 25 – Proportions and prevalence ratios of any cancer screening in the past 12 months in body mass index categories with alternative categorization of body mass index among older men and women above 75 years of age (weighted) 44](#_Toc207095831)

[Appendix Table 26 – Proportions of any cancer screening in the past 12 months in granular body mass index categories among older adults above 75 years of age (unweighted) 45](#_Toc207095832)

[Appendix Table 27 – Proportions and prevalence ratios of any cancer screening in the past 12 months in body mass index categories with alternative categorization of body mass index among older adults above 75 years of age (unweighted) 46](#_Toc207095833)

[Appendix Table 28 – Proportions and prevalence ratios of any cancer screening in the past 12 months in body mass index categories with alternative categorization of body mass index among older men and women above 75 years of age (unweighted) 47](#_Toc207095834)

[Appendix Table 29 – Proportions of colorectal cancer screening in the past 12 months in granular body mass index categories among older adults above 75 years of age (weighted) 48](#_Toc207095835)

[Appendix Table 30 – Proportions and prevalence ratios of any colorectal cancer screening in the past 12 months in body mass index categories with alternative categorization of body mass index among older adults above 75 years of age (weighted) 49](#_Toc207095836)

[Appendix Table 31 – Proportions and prevalence ratios of FOBT and colonoscopy screening in the past 12 months in body mass index categories with alternative categorization of body mass index among older adults above 75 years of age (weighted) 50](#_Toc207095837)

[Appendix Table 32 – Proportions of colorectal cancer screening in the past 12 months in granular body mass index categories among older adults above 75 years of age (unweighted) 51](#_Toc207095838)

[Appendix Table 33 – Proportions and prevalence ratios of any colorectal cancer screening in the past 12 months in body mass index categories with alternative categorization of body mass index among older adults above 75 years of age (unweighted) 52](#_Toc207095839)

[Appendix Table 34 – Proportions and prevalence ratios of FOBT and colonoscopy screening in the past 12 months in body mass index categories with alternative categorization of body mass index among older adults above 75 years of age (unweighted) 53](#_Toc207095840)

[Appendix Table 35 – Proportions of breast, cervical, and prostate cancer screening in the past 12 months in granular body mass index categories among older adults above 75 years of age (weighted) 54](#_Toc207095841)

[Appendix Table 36 – Proportions and prevalence ratios of breast and cervical cancer screening in the past 12 months in body mass index categories with alternative categorization of body mass index among older women above 75 years of age (weighted) 55](#_Toc207095842)

[Appendix Table 37 – Proportions and prevalence ratios of prostate cancer screening in the past 12 months in body mass index categories with alternative categorization of body mass index among older men above 75 years of age (weighted) 56](#_Toc207095843)

[Appendix Table 38 – Proportions of breast, cervical, and prostate cancer screening in the past 12 months in granular body mass index categories among older adults above 75 years of age (unweighted) 57](#_Toc207095844)

[Appendix Table 39 – Proportions and prevalence ratios of breast and cervical cancer screening in the past 12 months in body mass index categories with alternative categorization of body mass index among older women above 75 years of age (unweighted) 58](#_Toc207095845)

[Appendix Table 40 – Proportions and prevalence ratios of prostate cancer screening in the past 12 months in body mass index categories with alternative categorization of body mass index among older men above 75 years of age (unweighted) 59](#_Toc207095846)

[Supplemental material references 60](#_Toc207095847)

# Appendix Table 1 - Strengthening the reporting of observational studies in epidemiology (STROBE) checklist

|  | Item No | Recommendation | Location |
| --- | --- | --- | --- |
| **Title and abstract** | 1 | (*a*) Indicate the study’s design with a commonly used term in the title or the abstract | Abstract |
|  |  | (*b*) Provide in the abstract an informative and balanced summary of what was done and what was found | Abstract |
| Introduction | | |  |
| Background/rationale | 2 | Explain the scientific background and rationale for the investigation being reported | Introduction |
| Objectives | 3 | State specific objectives, including any prespecified hypotheses | Abstract; Introduction |
| Methods | | |  |
| Study design | 4 | Present key elements of study design early in the paper | Study design and data source |
| Setting | 5 | Describe the setting, locations, and relevant dates, including periods of recruitment, exposure, follow-up, and data collection | Study design and data source; Population |
| Participants | 6 | (*a*) Give the eligibility criteria, and the sources and methods of selection of participants | Study design and data source; Study population; Figure S1 |
| Variables | 7 | Clearly define all outcomes, exposures, predictors, potential confounders, and effect modifiers. Give diagnostic criteria, if applicable | Variables of interest; Tables S3-S5 |
| Data sources/ measurement | 8* | For each variable of interest, give sources of data and details of methods of assessment (measurement). Describe comparability of assessment methods if there is more than one group | Variables of interest; Tables S3-S5 |
| Bias | 9 | Describe any efforts to address potential sources of bias | Statistical analysis |
| Study size | 10 | Explain how the study size was arrived at | Study design and data source; Study population |
| Quantitative variables | 11 | Explain how quantitative variables were handled in the analyses. If applicable, describe which groupings were chosen and why | Variables of interest; Tables S3-S5 |
| Statistical methods | 12 | (*a*) Describe all statistical methods, including those used to control for confounding | Statistical analysis |
|  |  | (*b*) Describe any methods used to examine subgroups and interactions | Statistical analysis |
|  |  | (*c*) Explain how missing data were addressed | Study population |
|  |  | (*d*) If applicable, describe analytical methods taking account of sampling strategy | Statistical analysis |
|  |  | (*e*) Describe any sensitivity analyses | Sensitivity analysis |
| Results | | |  |
| Participants | 13* | (a) Report numbers of individuals at each stage of study—eg numbers potentially eligible, examined for eligibility, confirmed eligible, included in the study, completing follow-up, and analysed | Data source; Study population; Figure S1 |
|  |  | (b) Give reasons for non-participation at each stage | Figure S1 |
|  |  | (c) Consider use of a flow diagram | Figure S1 |
| Descriptive data | 14* | (a) Give characteristics of study participants (eg demographic, clinical, social) and information on exposures and potential confounders | Table 1 |
|  |  | (b) Indicate number of participants with missing data for each variable of interest | Study population; Table S2 |
| Outcome data | 15* | Report numbers of outcome events or summary measures | Tables 2 and S7 |
| Main results | 16 | (*a*) Give unadjusted estimates and, if applicable, confounder-adjusted estimates and their precision (eg, 95% confidence interval). Make clear which confounders were adjusted for and why they were included | Tables 3-4 and S8-S17 |
|  |  | (*b*) Report category boundaries when continuous variables were categorized | Variables of interest; Tables S3-S5 |
|  |  | (*c*) If relevant, consider translating estimates of relative risk into absolute risk for a meaningful time period | N/A |
| Other analyses | 17 | Report other analyses done—eg analyses of subgroups and interactions, and sensitivity analyses | Tables S18-S40 |
| Discussion | | |  |
| Key results | 18 | Summarise key results with reference to study objectives | Discussion |
| Limitations | 19 | Discuss limitations of the study, taking into account sources of potential bias or imprecision. Discuss both direction and magnitude of any potential bias | Discussion |
| Interpretation | 20 | Give a cautious overall interpretation of results considering objectives, limitations, multiplicity of analyses, results from similar studies, and other relevant evidence | Discussion |
| Generalisability | 21 | Discuss the generalisability (external validity) of the study results | Discussion |
| Other information | | |  |
| Funding | 22 | Give the source of funding and the role of the funders for the present study and, if applicable, for the original study on which the present article is based | Footnotes |

# Appendix Figure 1 – Flowchart of analytical sample inclusion


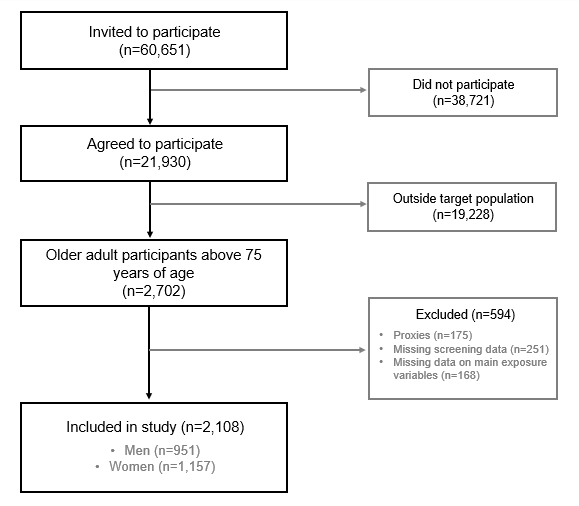


# Appendix Table 2 – Missing data for each variable of interest among all non-proxy respondents of the Swiss Health Survey above 75 years of age

| **Variable** | **Number missing** |
| --- | --- |
| **Screening variables** | |
| FOBT yes/no | 59 |
| FOBT reason | 26 |
| FOBT timing | 15 |
| Colonoscopy yes/no | 35 |
| Colonoscopy reason | 34 |
| Colonoscopy timing | 12 |
| Mammography yes/no | 3 |
| Mammography reason | 18 |
| Mammography timing | 21 |
| Cervical smear yes/no | 45 |
| Cervical smear timing | 18 |
| PSA or rectal exam yes/no | 9 |
| PSA or rectal exam reason | 19 |
| PSA or rectal exam timing | 10 |
| **Health status variables** | |
| Self-rated health | 6 |
| Chronic condition or long-term health issue | 14 |
| Number of morbidities*  Asthma  Pulmonary disorders  Hypertension  Elevated cholesterol  Diabetes  Myocardial infarction  Stroke  Cancer  Depression | 7  11  38  189  146  2  8  10  9 |
| Number of medications*  Hypertension medication  Heart medication  Insomnia medication  Pain medication  Calming medication  Attention medication  Cholesterol medication  Depression medication  Diabetes medication  Osteoporosis medication | 10  17  3  6  7  7  16  6  4  6 |
| Activities of daily living | 1 |
| Instrumental activities of daily living | 137 |
| Functional limitations | 18 |
| Smoking status | 2 |
| Body mass index | 47 |
| **Socio-demographic variables** | |
| Age | 0 |
| Sex | 0 |
| Education level | 24 |
| Linguistic region | 0 |

Abbreviations: Fecal occult blood test, FOBT; prostate specific antigen test, PSA.

* Participants were not excluded for having missing data for individual morbidities/medications. Instead, morbidities/medications with missing data were counted as null for the sum of morbidities/medications.

# Appendix Table 3 – Ascertainment of health status variables

| **Variable** | **Ascertainment method** |
| --- | --- |
| Self-rated health | “How is your overall state of health? Is it:”   - Very good - Good - Average - Bad - Very bad   We collapsed this variable into:   - Good or very good - Average - Bad or very bad |
| Chronic condition or long-term health issue | “Do you have an illness or health issue that is chronic of a long duration? By this we mean an illness or health issue that last at least 6 months or that will probably last 6 or more months.”   - Yes - No |
| Number of morbidities | Asthma, pulmonary disorders, and depression  “Over the course of the last 12 months, have you had any of the following illnesses or health issues?”   - Options relevant to this variable we constructed:   - Asthma   - Bronchitis, chronic obstructive pulmonary disease, or emphysema   - Depression - Participants could respond:   - Yes   - No   Myocardial infarction, stroke, and cancer  “Over the course of your life, have you ever had one of the following illnesses or health issues?   1. Myocardial infarction (heart attack) 2. Stroke (cerebral haemorrhage or thrombosis) 3. Cancer”   Participants could respond:   - Yes - No   Hypertension  Constructed by the Federal Office of Statistics. Older adults above 75 were classified as having hypertension if they either:   1. Answered as “too elevated” for the question: “Currently, is your arterial pressure normal, too low, or too elevated?”    - Normal    - Too low    - Too elevated 2. Answered “every day”, “multiple times”, or “around 1 time over the last 7 days” to the question: “At which frequency have you taken the following medication over the course of the last 7 days:    1. Medication against hypertension”       - Every day       - Multiple times       - Around 1 time over the last 7 days       - Never   Elevated cholesterol  Constructed by the Federal Office of Statistics. Older adults above 75 were classified as having elevated cholesterol if they either:   1. Answered as “too elevated” for the question: “Currently, is your cholesterol level normal or too elevated?”    - Normal    - Too elevated 2. Answered “every day”, “multiple times”, or “around 1 time over the last 7 days” to the question: “At which frequency have you taken the following medication over the course of the last 7 days:    1. Medication against cholesterol”       - Every day       - Multiple times       - Around 1 time over the last 7 days       - Never   Diabetes  Constructed by the Federal Office of Statistics. Older adults above 75 were classified as having diabetes if they either:   1. Answered as “too elevated” for the question: “Currently, is your glucose level (level of sugar in your blood) normal or too elevated?”    - Normal    - Too elevated 2. Answered “every day”, “multiple times”, or “around 1 time over the last 7 days” to the question: “At which frequency have you taken the following medication over the course of the last 7 days:    1. Medication against diabetes or insulin injection”       - Every day       - Multiple times       - Around 1 time over the last 7 days       - Never |
| Number of medications used | “I am going to read out a list of medications. At which frequency have you taken the following medication over the course of the last 7 days?   1. Medication against hypertension (against pressure) 2. Medication for the heart 3. Insomnia medication (e.g., Imovane, Sonata, Stilnox) 4. Medication against pain 5. Calming medication, tranquilisers (e.g., Valium, Xanax, Temesta, Lexotanil) 6. Medication for reinforcing attention or for staying awake (e.g., Ritalin, Medikinet, Concerta, Modasomil) 7. Medication against cholesterol 8. Medication against depression (e.g., Effexor, Fluoxétine, Deroxat) 9. Medication against diabetes or injecting insulin 10. Medication against osteoporosis”   For each medication, participants could answer either:   - Every day - Multiple times - Around 1 time over the last 7 days - Never |
| Difficulties with activities of daily living | Constructed by the Federal Office of Statistics based on the following question:   - “Tell me, for each of the following activity, whether you can complete them without difficulties, with some difficulties, with a lot of difficulties or not at all:  1. Eat without aid 2. Go to bed, get out of bed, getting up from the couch, without aid 3. Get dressed and undressed without aid 4. Go to the bathroom without aid 5. Take a bath or a shower without aid”  - For each activity, participants could respond either:   - Yes, without difficulties   - Yes, with some difficulties   - Yes, but with significant difficulties   - No   Constructed categories:   - No difficulty (if participants answered “Yes, without difficulties” for every question) - Some difficulty (if participants answered “Yes, with some difficulties” to at least one question) - A lot of difficulty (if participants answered “Yes, but with significant difficulties” to at least question) - Incapacitated (if participants answered “No” to at least one question)   Collapsed into:   - No difficulty - Difficulty (some difficulty, a lot of difficulty, and incapacitated) |
| Difficulties with instrumental activities of daily living | Constructed by the Federal Office of Statistics based on the following question:   - “Tell me, for each of the following activity, whether you can complete them without difficulties, with some difficulties, with a lot of difficulties or not at all:  1. Prepare meals without aid 2. Telephone without aid 3. Make purchases without aid 4. Do the dishes without aid 5. Do small household chores without aid 6. Do occasionally big household chores without aid 7. Make your accounts without aid 8. Utilise public transport without aid”  - For each activity, participants could respond either:   - Yes, without difficulties   - Yes, with some difficulties   - Yes, but with significant difficulties   - No   Constructed categories:   - No difficulty (if participants answered “Yes, without difficulties” to all questions) - Some difficulty (if participants answered “Yes, with some difficulties” to any question) - A lot of difficulty (if participants answered “Yes, but with significant difficulties” to at least one question) - Incapacitated (if participants answered “No” to at least one question)   Collapsed into:   - No difficulty - Difficulty (some difficulty, a lot of difficulty, and incapacitated) |
| Functional limitations | Constructed by the Federal Office of Statistics based on the following questions:   1. “Do you were correctives glasses or contact lenses?”    - Yes    - No    - I am blind 2. “Do you see well enough to read a book or journal?”    - Yes, without difficulties    - Yes, with some difficulties    - Yes, but with significant difficulties    - No 3. “Can you follow a conversation in which there are at least 2 other persons?”    - Yes, without difficulties    - Yes, with some difficulties    - Yes, but with significant difficulties    - No. 4. “For which distance can you walk by yourself without aid, without needing to stop, and without being significant inconvenienced.”    - 200 meters or more    - More than a few steps, but less than 200 meters    - Only a few steps    - I cannot move around 5. “Can you speak without difficulties?”    - Yes, without difficulties    - Yes, with some difficulties    - Yes, but with significant difficulties    - No   Constructed categories:   - No difficulty (answered “Yes, without difficulties” to questions 2,3,5 and “200 meters or more” to question 4) - Some difficulty (answered “Yes, with some difficulties” to at least one of questions 2,3,5 or “More than a few steps, but less than 200 meters” to question 4) - A lot of difficulty (answered “Yes, but with significant difficulties” to at least one of questions 2,3,5 or “Only a few steps” to question 4) - Incapacitated (answered “No” to at least one of questions 2,3,5 or “I cannot move around” to question 4 or “I am blind” to question 1)   Collapsed into:   - No difficulty - Difficulty (some difficulty, a lot of difficulty, and incapacitated) |
| Smoking status | Constructed by the Federal Office of Statistics based on the following questions:   1. “Do you smoke, even occasionally?”    1. Yes    2. No 2. For current non-smokers: “Have you smoked for more than 6 months regularly?”    - Yes    - No   Categorised into:   - Never smoker - Former smoker - Current smoker |
| Body mass index | Calculated by the Federal Office of Statistics based on the following questions:   1. “Could you indicate your height without shoes?” 2. “How much do you weigh without clothes?”   Categorised into:   - Not obese (BMI < 30) - Obese (30 <= BMI) |

# Appendix Table 4 – Ascertainment of cancer screening variables

| **Variable** | **Ascertainment method** |
| --- | --- |
| Colorectal cancer screening (fecal occult blood test) | “Have you undergone a test to detect blood in faeces invisible to the eye known as screening test for cancer of the intestines?”   - Yes - No   If participant answered yes:  “When did you undergo this test the last time?”   - Within the last 12 months - 1 year to less than 2 years ago - 2 years to less than 3 years ago - 3 years to less than 5 years ago - More than 5 years ago   “The last time, what was the reason you underwent this test? Was it:”   - For preventive non-symptomatic purposes - For diagnostic purposes following pain or symptoms - For diagnostic purposes following the results of a prior exam - For other reason |
| Colorectal cancer screening (colonoscopy) | “Have you undergone a colonoscopy? It is a visual exam of your big intestine by a thin metal probe.”  If participant answered yes:  “When did you undergo this colonoscopy for the last time?”   - Within the last 12 months - 1 year to less than 5 years ago - 5 years to less than 10 years ago - More than 10 years ago   “The last time, what was the reason you underwent this colonoscopy? Was it:”   - For preventive non-symptomatic purposes - For diagnostic purposes following pain or symptoms - For diagnostic purposes following the results of a prior exam - For other reason |
| Breast cancer screening (mammography) | “Have you undergone a mammogram?”   - Yes - No   If participant answered yes:  “When was you undergo a mammogram for the last time?”   - Within the last 12 months - 1 year to less than 2 years ago - 2 years to less than 3 years ago - 3 years to less than 5 years ago - More than 5 years ago   “The last time, what was the reason you underwent this mammogram? Was it:”   - For preventive non-symptomatic purposes - For diagnostic purposes following pain or symptoms - For diagnostic purposes following the results of a prior exam - For other reason |
| Cervical cancer screening (cervical smear) | “Have you undergone a cervical smear (vaginal smear)”?   - Yes - No   If participant answered yes:  “When did you a cervical smear (vaginal smear) for the last time?”   - Within the last 12 months - 1 year to less than 2 years ago - 2 years to less than 3 years ago - 3 years to less than 5 years ago - More than 5 years ago |
| Prostate cancer screening (prostate specific antigen test or rectal exam) | “Have you undergone a prostate exam?”   - Yes - No   If participant answered yes:  “How was this exam practiced the last time?”   - By rectal exam - By blood test (prostate specific antigen test) - By both   “What was the reason you underwent this exam the last time? Was it:”   - For preventive non-symptomatic purposes - For diagnostic purposes following pain or symptoms - For diagnostic purposes following the results of a prior exam - For other reason   “When did you undergo a prostate exam for the last time?”   - Within the last 12 months - 1 year to less than 2 years ago - 2 years to less than 3 years ago - 3 years to less than 5 years ago - More than 5 years ago |

# Appendix Table 5 – Ascertainment of socio-demographic variables

| **Variable** | **Ascertainment method** |
| --- | --- |
| Age | Self-reported date of birth.  Categorised into:   - 76-80 - 81-85 - 86+ |
| Sex | “Who are you?”   - Man - Woman |
| Education level | “What is the highest level of training that you have achieved?”  Coded into the following categories according to the International Standard Classification of Education by the Federal Office of Statistics:   - Obligatory schooling - Secondary degree - Tertiary degree |
| Linguistic region | Constructed by the Federal Office of Statistics using participants’ commune of residence of Switzerland.  Categorised into:   - German - French - Italian |

# Appendix Table 6 – Inclusion justifications for health status indicators

| **Variable** | **Justification for inclusion** |
| --- | --- |
| Self-rated health | Has been shown to be associated with life expectancy in results stratified by age with strata for older adults: (Cho et al, 2022) |
| Chronic condition or long-term health issue / number of morbidities | Chronic conditions and comorbidities are commonly found within life expectancy estimators and mortality indices for older adults: (Cho et al, 2022; Cruz et al, 2013; Gastens et al, 2025; Kobayashi et al, 2016; Yourman et al, 2013) |
| Number of medications used | Included in life expectancy estimator for older adults: (Gastens et al, 2025) |
| Difficulties with activities of daily living | Functional status is commonly included within mortality indices, including activities of daily living specifically: (Yourman et al, 2013). Moreover, variations of the following component of the activities of daily living variable (see Table S3) are included in mortality indices for older adults_   - Difficulties bathing (Cruz et al, 2013) |
| Difficulties with instrumental activities of daily living | Functional status is commonly included within mortality indices: (Yourman et al, 2013). Moreover, variations of the following components of the instrumental activities of daily living variable (see Table S3) are included in mortality indices for older adults:   - Difficulty preparing meals (Kobayashi et al, 2016) - Difficulties managing finances (Cruz et al, 2013) |
| Functional limitations | Functional status is commonly included within mortality indices: (Yourman et al, 2013). Moreover, variations of the following components of the instrumental activities of daily living variable (see Table S3) are included in mortality indices for older adults:   - Difficulty walking several blocks (Cruz et al, 2013) - Difficulty walking 100 yards (Kobayashi et al, 2016) |
| Smoking status | Included in mortality indices for older adults: (Cruz et al, 2013; Kobayashi et al, 2016) |
| Body mass index | Included in life expectancy estimator and mortality index for older adults: (Cruz et al, 2013; Gastens et al, 2025) |

# Appendix Table 7 – Proportions of any cancer screening in the past 12 months among older adults above 75 years of age (unweighted)

| **Cancer screening** | **All**  **(n=2,108)** | **Men**  **(n=951)** | **Women**  **(n=1,157)** |
| --- | --- | --- | --- |
| **Any cancer screening**  Yes  No | 539 (25.6%)  1,569 (74.4%) | 287 (30.2%)  664 (69.8%) | 252 (21.8%)  905 (78.2%) |
| **Colorectal cancer screening (FOBT or colonoscopy)**  Yes  No | 194 (9.2%)  1,914 (90.8%) | 125 (13.1%)  826 (86.9%) | 69 (6.0%)  1,088 (94.0%) |
| **Breast cancer screening (mammography)**  Yes  No | -  - | -  - | 71 (6.1%)  1,086 (93.9%) |
| **Cervical cancer screening (cervical smear)**  Yes  No | -  - | -  - | 179 (15.5%)  978 (84.5%) |
| **Prostate cancer screening (PSA or rectal exam)**  Yes  No | -  - | 209 (22.0%)  742 (78.0%) | -  - |

Abbreviations: Fecal occult blood test, FOBT; prostate specific antigen test, PSA.

# Appendix Table 8 – Proportions and prevalence ratios of any cancer screening in the past 12 months in categories of health status indicators and age group among all older adults above 75 years of age (unweighted)

|  | **All older adults (n = 2,108)** | | |
| --- | --- | --- | --- |
| **Screening according to health indicators** | **Proportions** | **PR (unadjusted)** | **aPR (adjusted for age and sex)** |
| **Age group**  76-80  81-85  86+ | 354 (30.4%)  143 (22.4%)  42 (13.8%) | (ref)  0.74 (0.62-0.87)  0.46 (0.34-0.61) | **-**  **-**  **-** |
| **Self-rated health**  Good or very good  Average  Bad or very bad | 395 (25.8%)  119 (24.2%)  25 (28.7%) | (ref)  0.94 (0.79-1.12)  1.11 (0.79-1.57) | (ref)  0.99 (0.83-1.18)  1.18 (0.84-1.65) |
| **Chronic condition or long-term health issue**  No  Yes | 242 (24.6%)  297 (26.4%) | (ref)  1.08 (0.93-1.25) | (ref)  1.08 (0.94-1.25) |
| **Number of morbidities***  0  1  2-3  4 or more | 91 (19.3%)  150 (23.6%)  244 (29.0%)  54 (33.8%) | (ref)  1.22 (0.97-1.54)  1.50 (1.21-1.86)  1.75 (1.31-2.32) | (ref)  1.25 (0.99-1.57)  1.47 (1.19-1.81)  1.68 (1.27-2.23) |
| **Number of medications****  0  1-2  3-4  5 or more | 84 (19.0%)  245 (25.4%)  180 (29.9%)  30 (30.3%) | (ref)  1.34 (1.08-1.67)  1.57 (1.25-1.98)  1.60 (1.12-2.28) | (ref)  1.37 (1.10-1.70)  1.61 (1.28-2.02)  1.70 (1.19-2.43) |
| **Activities of daily living**  No difficulty  Difficulty | 500 (25.8%)  39 (23.1%) | (ref)  0.89 (0.67-1.19) | (ref)  1.06 (0.80-1.42) |
| **Instrumental activities of daily living**  No difficulty  Difficulty | 354 (27.9%)  185 (22.0%) | (ref)  0.79 (0.67-0.92) | (ref)  0.91 (0.78-1.07) |
| **Functional limitations**  No difficulty  Difficulty | 402 (26.9%)  137 (22.3%) | (ref)  0.83 (0.70-0.98) | (ref)  0.90 (0.76-1.07) |
| **Smoking status**  Never smoker  Former smoker  Current smoker | 263 (22.8%)  231 (30.6%)  45 (22.7%) | (ref)  1.34 (1.16-1.56)  1.00 (0.76-1.32) | (ref)  1.20 (1.03-1.41)  0.90 (0.68-1.18) |
| **Body mass index**  Not obese  Obese | 464 (25.4%)  75 (26.8%) | (ref)  1.06 (0.86-1.30) | (ref)  1.00 (0.82-1.23) |

Abbreviations: PR, prevalence ratio; aPR, adjusted prevalence ratio.

* based on 9 different morbidities: asthma, pulmonary disorders (bronchitis, COPD, and emphysema), hypertension, elevated cholesterol, diabetes, myocardial infarction, stroke, cancer, and depression.

** based on 10 different medication types: hypertension medication, heart medication, insomnia medication, pain medication, calming medication, attention medication, cholesterol medication, depression medication, diabetes medication, and osteoporosis medication.

# Appendix Table 9 – Proportions and prevalence ratios of any cancer screening in the past 12 months in categories of health status indicators and age group among older men and women above 75 years of age (unweighted)

|  | **Men (n=951)** | | | **Women (n=1,157)** | | |
| --- | --- | --- | --- | --- | --- | --- |
| **Screening according to health indicators** | **Proportions** | **PR (unadjusted)** | **aPR (adjusted for age and sex)** | **Proportions** | **PR (unadjusted)** | **aPR (adjusted for age and sex)** |
| **Age group**  76-80  81-85  86+ | 182 (34.0%)  78 (27.1%)  27 (21.3%) | (ref)  0.80 (0.64-1.00)  (0.63 (0.44-0.89) | **-**  **-**  **-** | 172 (27.3%)  65 (18.6%)  15 (8.5%) | (ref)  0.68 (0.53-0.88)  0.31 (0.19-0.51) | **-**  **-**  **-** |
| **Self-rated health**  Good or very good  Average  Bad or very bad | 210 (29.0%)  66 (34.2%)  11 (31.4%) | (ref)  1.18 (0.94-1.48)  1.08 (0.65-1.79) | (ref)  1.16 (0.93-1.46)  1.10 (0.67-1.81) | 185 (22.9%)  53 (17.8%)  14 (26.9%) | (ref)  0.78 (0.59-1.02)  1.17 (0.74-1.87) | (ref)  0.84 (0.64-1.11)  1.24 (0.79-1.94) |
| **Chronic condition or long-term health issue**  No  Yes | 122 (27.5%)  165 (32.5%) | (ref)  1.18 (0.97-1.44) | (ref)  1.18 (0.97-1.43) | 120 (22.2%)  132 (21.4%) | (ref)  0.97 (0.78-1.20) | (ref)  1.00 (0.80-1.24) |
| **Number of morbidities***  0  1  2-3  4 or more | 38 (20.2%)  69 (27.4%)  143 (34.0%)  37 (41.1%) | (ref)  1.35 (0.96-1.92)  1.68 (1.23-2.30)  2.03 (1.40-2.96) | (ref)  1.36 (0.96-1.92)  1.65 (1.21-2.26)  1.99 (1.37-2.90) | 53 (18.7%)  81 (21.1%)  101 (24.0%)  17 (24.3%) | (ref)  1.13 (0.83-1.54)  1.28 (0.95-1.72)  1.30 (0.80-2.10) | (ref)  1.18 (0.87-1.59)  1.36 (1.02-1.82)  1.41 (0.87-2.28) |
| **Number of medications****  0  1-2  3-4  5 or more | 40 (20.2%)  128 (29.6%)  106 (37.2%)  13 (37.1%) | (ref)  1.46 (1.07-2.00)  1.84 (1.34-2.52)  1.84 (1.10-3.07) | (ref)  1.49 (1.09-2.03)  1.86 (1.36-2.54)  1.85 (1.10-3.11) | 44 (18.0%)  117 (22.1%)  74 (23.3%)  17 (26.6%) | (ref)  1.23 (0.90-1.68)  1.30 (0.93-1.81)  1.48 (0.91-2.41) | (ref)  1.25 (0.92-1.70)  1.39 (1.00-1.93)  1.59 (0.98-2.58) |
| **Activities of daily living**  No difficulty  Difficulty | 269 (30.4%)  18 (27.3%) | (ref)  0.90 (0.60-1.35) | (ref)  0.95 (0.63-1.44) | 231 (21.9%)  21 (20.4%) | (ref)  0.93 (0.62-1.38) | (ref)  1.24 (0.83-1.84) |
| **Instrumental activities of daily living**  No difficulty  Difficulty | 205 (31.5%)  82 (27.3%) | (ref)  0.87 (0.70-1.08) | (ref)  0.92 (0.74-1.14) | 149 (24.2%)  103 (19.0%) | (ref)  0.79 (0.63-0.98) | (ref)  0.93 (0.74-1.16) |
| **Functional limitations**  No difficulty  Difficulty | 214 (31.4%)  73 (27.0%) | (ref)  0.86 (0.69-1.08) | (ref)  0.90 (0.72-1.13) | 188 (23.1%)  64 (18.7%) | (ref)  0.81 (0.63-1.04) | (ref)  0.91 (0.71-1.17) |
| **Smoking status**  Never smoker  Former smoker  Current smoker | 109 (28.1%)  145 (31.3%)  33 (33.0%) | (ref)  1.11 (0.90-1.37)  1.17 (0.85-1.62) | (ref)  1.09 (0.89-1.34)  1.12 (0.81-1.54) | 154 (20.1%)  86 (29.5%)  12 (12.2%) | (ref)  1.47 (1.17-1.84)  0.61 (0.35-1.06) | (ref)  1.39 (1.11-1.74)  0.58 (0.34-1.00) |
| **Body mass index**  Not obese  Obese | 238 (28.9%)  49 (38.3%) | (ref)  1.32 (1.04-1.69) | (ref)  1.26 (0.99-1.62) | 226 (22.5%)  26 (17.1%) | (ref)  0.76 (0.53-1.10) | (ref)  0.73 (0.51-1.05) |

Abbreviations: PR, prevalence ratio; aPR, adjusted prevalence ratio.

* based on 9 different morbidities: asthma, pulmonary disorders (bronchitis, COPD, and emphysema), hypertension, elevated cholesterol, diabetes, myocardial infarction, stroke, cancer, and depression.

** based on 10 different medication types: hypertension medication, heart medication, insomnia medication, pain medication, calming medication, attention medication, cholesterol medication, depression medication, diabetes medication, and osteoporosis medication.

# Appendix Table 10 – Proportions and prevalence ratios of any colorectal cancer screening in the past 12 months in categories of health status indicators and age group among all older adults above 75 years of age (weighted)

|  | **Any colorectal (all older adults; n = 2,108)** | | |
| --- | --- | --- | --- |
| **Screening according to health indicators** | **Proportions** | **PR (unadjusted)** | **aPR (adjusted for age and sex)** |
| **Age group**  76-80  81-85  86+ | 12.3%  6.3%  6.4% | (ref)  0.51 (0.34-0.76)  0.52 (0.31-0.89) | -  -  - |
| **Self-rated health**  Good or very good  Average  Bad or very bad | 9.1%  10.2%  8.0% | (ref)  1.12 (0.76-1.64)  0.87 (0.40-1.92) | (ref)  1.20 (0.82-1.76)  0.97 (0.45-2.06) |
| **Chronic condition or long-term health issue**  No  Yes | 8.4%  10.2% | (ref)  1.21 (0.88-1.66) | (ref)  1.24 (0.91-1.70) |
| **Number of morbidities***  0  1  2-3  4 or more | 7.0%  7.6%  10.7%  15.3% | (ref)  1.09 (0.66-1.82)  1.54 (0.98-2.43)  2.20 (1.27-3.82) | (ref)  1.11 (0.67-1.83)  1.47 (0.93-2.30)  2.03 (1.17-3.53) |
| **Number of medications****  0  1-2  3-4  5 or more | 7.6%  8.3%  11.7%  13.0% | (ref)  1.09 (0.69-1.73)  1.54 (0.96-2.47)  1.71 (0.88-3.32) | (ref)  1.09 (0.69-1.73)  1.56 (0.98-2.49)  1.86 (0.96-3.61) |
| **Activities of daily living**  No difficulty  Difficulty | 9.7%  5.4% | (ref)  0.55 (0.29-1.06) | (ref)  0.64 (0.33-1.24) |
| **Instrumental activities of daily living**  No difficulty  Difficulty | 11.1%  6.9% | (ref)  0.62 (0.44-0.87) | (ref)  0.75 (0.52-1.07) |
| **Functional limitations**  No difficulty  Difficulty | 10.3%  7.1% | (ref)  0.68 (0.47-0.99) | (ref)  0.75 (0.52-1.08) |
| **Smoking status**  Never smoker  Former smoker  Current smoker | 8.5%  10.4%  10.3% | (ref)  1.22 (0.87-1.71)  1.21 (0.72-2.05) | (ref)   - 1. (0.72-1.42)   1.01 (0.61-1.69) |
| **Body mass index**  Not obese  Obese | 9.1%  11.0% | (ref)  1.21 (0.76-1.94) | (ref)  1.14 (0.71-1.84) |

Abbreviations: PR, prevalence ratio; aPR, adjusted prevalence ratio.

* based on 9 different morbidities: asthma, pulmonary disorders (bronchitis, COPD, and emphysema), hypertension, elevated cholesterol, diabetes, myocardial infarction, stroke, cancer, and depression.

** based on 10 different medication types: hypertension medication, heart medication, insomnia medication, pain medication, calming medication, attention medication, cholesterol medication, depression medication, diabetes medication, and osteoporosis medication.

# Appendix Table 11 – Proportions and prevalence ratios of any colorectal cancer screening in the past 12 months in categories of health status indicators and age group among all older adults above 75 years of age (unweighted)

|  | **Any colorectal (all older adults; n = 2,108)** | | |
| --- | --- | --- | --- |
| **Screening according to health indicators** | **Proportions** | **PR (unadjusted)** | **aPR (adjusted for age and sex)** |
| **Age group**  76-80  81-85  86+ | 132 (11.3%)  42 (6.6%)  20 (6.6%) | (ref)  0.58 (0.42-0.81)  0.58 (0.37-0.91) | -  -  - |
| **Self-rated health**  Good or very good  Average  Bad or very bad | 141 (9.2%)  45 (9.2%)  8 (9.2%) | (ref)  0.99 (0.72-1.37)  1.00 (0.51-1.97) | (ref)  1.07 (0.78-1.47)  1.09 (0.55-2.10) |
| **Chronic condition or long-term health issue**  No  Yes | 85 (8.6%)  109 (9.7%) | (ref)  1.12 (0.86-1.47) | (ref)  1.13 (0.86-1.47) |
| **Number of morbidities***  0  1  2-3  4 or more | 30 (6.4%)  48 (7.6%)  88 (10.5%)  28 (17.5%) | (ref)  1.19 (0.76-1.84)  1.64 (1.10-2.44)  2.75 (1.70-4.45) | (ref)  1.20 (0.78-1.86)  1.53 (1.03-2.26)  2.45 (1.51-3.96) |
| **Number of medications****  0  1-2  3-4  5 or more | 31 (7.0%)  83 (8.6%)  65 (10.8%)  15 (15.2%) | (ref)  1.23 (0.83-1.83)  1.54 (1.02-2.32)  2.17 (1.22-3.85) | (ref)  1.25 (0.84-1.86)  1.55 (1.03-2.32)  2.39 (1.36-4.23) |
| **Activities of daily living**  No difficulty  Difficulty | 181 (9.3%)  13 (7.7%) | (ref)  0.82 (0.48-1.41) | (ref)  0.98 (0.57-1.68) |
| **Instrumental activities of daily living**  No difficulty  Difficulty | 133 (10.5%)  61 (7.3%) | (ref)  0.69 (0.52-0.92) | (ref)  0.85 (0.63-1.15) |
| **Functional limitations**  No difficulty  Difficulty | 148 (9.9%)  46 (7.5%) | (ref)  0.76 (0.55-1.04) | (ref)  0.82 (0.60-1.12) |
| **Smoking status**  Never smoker  Former smoker  Current smoker | 88 (7.6%)  84 (11.1%)  22 (11.1%) | (ref)  1.46 (1.10-1.94)  1.46 (0.94-2.27) | (ref)  1.16 (0.86-1.56)  1.21 (0.78-1.87) |
| **Body mass index**  Not obese  Obese | 165 (9.0%)  29 (10.4%) | (ref)  1.15 (0.79-1.67) | (ref)  1.09 (0.75-1.57) |

Abbreviations: PR, prevalence ratio; aPR, adjusted prevalence ratio.

* based on 9 different morbidities: asthma, pulmonary disorders (bronchitis, COPD, and emphysema), hypertension, elevated cholesterol, diabetes, myocardial infarction, stroke, cancer, and depression.

** based on 10 different medication types: hypertension medication, heart medication, insomnia medication, pain medication, calming medication, attention medication, cholesterol medication, depression medication, diabetes medication, and osteoporosis medication.

# Appendix Table 12 – Proportions and prevalence ratios of FOBT and colonoscopy screening in the past 12 months in categories of health status indicators and age group among all older adults above 75 years of age (weighted)

|  | **FOBT (all older adults; n = 2,108)** | | | **Colonoscopy (all older adults; n = 2,108)** | | |
| --- | --- | --- | --- | --- | --- | --- |
| **Screening according to health indicators** | **Proportions** | **PR (unadjusted)** | **aPR (adjusted for age and sex)** | **Proportions** | **PR (unadjusted)** | **aPR (adjusted for age and sex)** |
| **Age group**  76-80  81-85  86+ | 6.6%  3.7%  4.7% | (ref)  0.57 (0.34-0.94)  0.71 (0.37-1.37) | -  -  - | 7.7%  3.4%  2.6% | (ref)  0.44 (0.25-0.78)  0.34 (0.15-0.80) | -  -  - |
| **Self-rated health**  Good or very good  Average  Bad or very bad | 4.9%  6.3%  7.5% | (ref)  1.27 (0.75-2.14)  1.52 (0.65-3.53) | (ref)  1.37 (0.81-2.31)  1.66 (0.73-3.81) | 5.6%  5.6%  2.7% | (ref)   - 1. (0.58-1.74)   0.49 (0.16-1.44) | (ref)  1.07 (0.63-1.83)  0.54 (0.18-1.58) |
| **Chronic condition or long-term health issue**  No  Yes | 4.8%  5.8% | (ref)  1.21 (0.78-1.87) | (ref)  1.24 (0.80-1.91) | 4.8%  6.0% | (ref)  1.26 (0.82-1.94) | (ref)  1.29 (0.85-1.97) |
| **Number of morbidities***  0  1  2-3  4 or more | 4.0%  3.9%  6.1%  10.3% | (ref)  0.98 (0.49-1.95)  1.53 (0.83-2.82)  2.58 (1.27-5.21) | (ref)  0.98 (0.50-1.92)  1.43 (0.78-2.65)  2.32 (1.14-4.72) | 4.4%  4.7%  6.2%  7.4% | (ref)  1.06 (0.54-2.08)  1.39 (0.76-2.53)  1.66 (0.76-3.63) | (ref)  1.09 (0.56-2.13)  1.36 (0.75-2.48)  1.62 (0.74-3.56) |
| **Number of medications****  0  1-2  3-4  5 or more | 4.2%  4.2%  7.3%  10.3% | (ref)  0.99 (0.52-1.89)  1.72 (0.91-3.25)  2.43 (1.07-5.52) | (ref)  0.98 (0.51-1.89)  1.72 (0.91-3.24)  2.61 (1.14-5.97) | 5.6%  5.4%  5.2%  6.9% | (ref)  0.97 (0.54-1.74)  0.94 (0.50-1.76)  1.24 (0.49-3.13) | (ref)  0.98 (0.55-1.77)  0.97 (0.52-1.82)  1.37 (0.54-3.45) |
| **Activities of daily living**  No difficulty  Difficulty | 5.6%  2.4% | (ref)  0.42 (0.15-1.15) | (ref)  0.46 (0.17-1.26) | 5.6%  4.2% | (ref)  0.76 (0.35-1.65) | (ref)  0.95 (0.43-2.10) |
| **Instrumental activities of daily living**  No difficulty  Difficulty | 6.3%  4.0% | (ref)  0.64 (0.40-1.02) | (ref)  0.75 (0.45-1.25) | 6.7%  3.7% | (ref)  0.55 (0.35-0.88) | (ref)  0.68 (0.42-1.08) |
| **Functional limitations**  No difficulty  Difficulty | 6.1%  3.6% | (ref)  0.60 (0.35-1.01) | (ref)  0.63 (0.37-1.06) | 5.8%  4.6% | (ref)  0.79 (0.49-1.29) | (ref)  0.90 (0.56-1.44) |
| **Smoking status**  Never smoker  Former smoker  Current smoker | 4.7%  6.4%  5.2% | (ref)  1.36 (0.86-2.14)  1.11 (0.51-2.42) | (ref)  1.11 (0.70-1.77)  0.94 (0.43-2.04) | 5.4%  5.7%  5.1% | (ref)  1.05 (0.67-1.66)  0.95 (0.46-1.97) | (ref)  0.91 (0.59-1.40)  0.80 (0.40-1.61) |
| **Body mass index**  Not obese  Obese | 5.0%  7.7% | (ref)  1.53 (0.82-2.84) | (ref)  1.49 (0.80-2.79) | 5.1%  7.7% | (ref)  1.51 (0.81-2.78) | (ref)  1.37 (0.73-2.56) |

Abbreviations: Fecal occult blood test, FOBT; PR, prevalence ratio; aPR, adjusted prevalence ratio.

* based on 9 different morbidities: asthma, pulmonary disorders (bronchitis, COPD, and emphysema), hypertension, elevated cholesterol, diabetes, myocardial infarction, stroke, cancer, and depression.

** based on 10 different medication types: hypertension medication, heart medication, insomnia medication, pain medication, calming medication, attention medication, cholesterol medication, depression medication, diabetes medication, and osteoporosis medication.

# Appendix Table 13 – Proportions and prevalence ratios of FOBT and colonoscopy screening in the past 12 months in categories of health status indicators and age group among all older adults above 75 years of age (unweighted)

|  | **FOBT (all older adults; n = 2,108)** | | | **Colonoscopy (all older adults; n = 2,108)** | | |
| --- | --- | --- | --- | --- | --- | --- |
| **Screening according to health indicators** | **Proportions** | **PR (unadjusted)** | **aPR (adjusted for age and sex)** | **Proportions** | **PR (unadjusted)** | **aPR (adjusted for age and sex)** |
| **Age group**  76-80  81-85  86+ | 68 (5.8%)  27 (4.2%)  14 (4.6%) | (ref)  0.73 (0.47-1.12)  0.79 (0.45-1.38) | -  -  - | 84 (7.2%)  21 (3.3%)  8 (2.6%) | (ref)  0.46 (0.29-0.73)  0.37 (0.18-0.75) | -  -  - |
| **Self-rated health**  Good or very good  Average  Bad or very bad | 75 (4.9%)  27 (5.5%)  7 (8.0%) | (ref)  1.12 (0.73-1.72)  1.64 (0.78-3.45) | (ref)  1.22 (0.79-1.87)  1.78 (0.86-3.70) | 87 (5.7%)  22 (4.5%)  4 (4.6%) | (ref)  0.79 (0.50-1.24)  0.81 (0.30-2.15) | (ref)  0.85 (0.54-1.34)  0.89 (0.34-2.33) |
| **Chronic condition or long-term health issue**  No  Yes | 46 (4.7%)  63 (5.6%) | (ref)  1.20 (0.83-1.74) | (ref)  1.20 (0.83-1.74) | 50 (5.1%)  63 (5.6%) | (ref)  1.11 (0.77-1.59) | (ref)  1.11 (0.78-1.60) |
| **Number of morbidities***  0  1  2-3  4 or more | 17 (3.6%)  25 (3.9%)  48 (5.7%)  19 (11.9%) | (ref)  1.09 (0.60-2.00)  1.58 (0.92-2.71)  3.29 (1.75-6.17) | (ref)  1.10 (0.60-2.01)  1.45 (0.85-2.47)  2.86 (1.53-5.35) | 18 (3.8%)  28 (4.4%)  54 (6.4%)  13 (8.2%) | (ref)  1.15 (0.65-2.06)  1.68 (1.00-2.83)  2.13 (1.07-4.24) | (ref)  1.18 (0.66-2.10)  1.60 (0.95-2.68)  1.95 (0.98-3.88) |
| **Number of medications****  0  1-2  3-4  5 or more | 17 (3.8%)  41 (4.3%)  40 (6.6%)  11 (11.1%) | (ref)  1.11 (0.64-1.93)  1.73 (0.99-3.01)  2.90 (1.40-5.99) | (ref)  1.12 (0.64-1.95)  1.71 (0.98-2.96)  3.21 (1.55-6.63) | 20 (4.5%)  53 (5.5%)  32 (5.3%)  8 (8.1%) | (ref)  1.22 (0.74-2.01)  1.18 (0.68-2.03)  1.79 (0.81-3.95) | (ref)  1.25 (0.76-2.06)  1.20 (0-70-2.07)  2.00 (0.91-4.38) |
| **Activities of daily living**  No difficulty  Difficulty | 103 (5.3%)  6 (3.6%) | (ref)  0.67 (0.30-1.50) | (ref)  0.76 (0.34-1.69) | 104 (5.4%)  9 (5.3%) | (ref)  0.99 (0.51-1.93) | (ref)  1.28 (0.66-2.48) |
| **Instrumental activities of daily living**  No difficulty  Difficulty | 76 (6.0%)  33 (3.9%) | (ref)  0.65 (0.44-0.97) | (ref)  0.79 (0.52-1.19) | 79 (6.2%)  34 (4.0%) | (ref)  0.65 (0.44-0.96) | (ref)  0.82 (0.55-1.23) |
| **Functional limitations**  No difficulty  Difficulty | 87 (5.8%)  22 (3.6%) | (ref)  0.62 (0.39-0.98) | (ref)  0.65 (0.41-1.02) | 84 (5.6%)  29 (4.7%) | (ref)  0.84 (0.56-1.27) | (ref)  0.94 (0.63-1.42) |
| **Smoking status**  Never smoker  Former smoker  Current smoker | 49 (4.2%)  50 (6.6%)  10 (5.1%) | (ref)  1.56 (1.06-2.29)  1.19 (0.61-2.31) | (ref)  1.21 (0.81-1.81)  0.99 (0.51-1.91) | 52 (4.5%)  49 (6.5%)  12 (6.1%) | (ref)  1.44 (0.99-2.11)  1.35 (0.73-2.48) | (ref)  1.17 (0.79-1.72)  1.11 (0.61-2.04) |
| **Body mass index**  Not obese  Obese | 91 (5.0%)  18 (6.4%) | (ref)  1.29 (0.79-2.11) | (ref)  1.25 (0.77-2.04) | 95 (5.2%)  18 (6.4%) | (ref)  1.24 (0.76-2.02) | (ref)  1.14 (0.70-1.86) |

Abbreviations: Fecal occult blood test, FOBT; PR, prevalence ratio; aPR, adjusted prevalence ratio.

* based on 9 different morbidities: asthma, pulmonary disorders (bronchitis, COPD, and emphysema), hypertension, elevated cholesterol, diabetes, myocardial infarction, stroke, cancer, and depression.

** based on 10 different medication types: hypertension medication, heart medication, insomnia medication, pain medication, calming medication, attention medication, cholesterol medication, depression medication, diabetes medication, and osteoporosis medication.

# Appendix Table 14 – Proportions and prevalence ratios of breast and cervical cancer screening in the past 12 months in categories of health status indicators and age group among older women above 75 years of age (weighted)

|  | **Mammography (women; n = 1,157)** | | | **Cervical smear (women; n = 1,157)** | | |
| --- | --- | --- | --- | --- | --- | --- |
| **Screening according to health indicators** | **Proportions** | **PR (unadjusted)** | **aPR (adjusted for age and sex)** | **Proportions** | **PR (unadjusted)** | **aPR (adjusted for age and sex)** |
| **Age group**  76-80  81-85  86+ | 7.8%  3.9%  1.8% | (ref)  0.50 (0.26-0.95)  0.24 (0.08-0.70) | -  -  - | 20.0%  11.4%  4.6% | (ref)  0.57 (0.40-0.83)  0.23 (0.10-0.54) | -  -  - |
| **Self-rated health**  Good or very good  Average  Bad or very bad | 5.5%  5.7%  4.4% | (ref)  1.04 (0.58-1.88)  0.81 (0.23-2.88) | (ref)  1.24 (0.68-2.24)  0.95 (0.28-3.23) | 15.6%  9.9%  22.3% | (ref)  0.63 (0.41-0.97)  1.43 (0.77-2.64) | (ref)  0.72 (0.47-1.10)  1.60 (0.89-2.87) |
| **Chronic condition or long-term health issue**  No  Yes | 5.0%  5.9% | (ref)  1.17 (0.71-1.95) | (ref)  1.23 (0.74-2.03) | 14.1%  14.6% | (ref)  1.04 (0.76-1.43) | (ref)  1.07 (0.78-1.48) |
| **Number of morbidities***  0  1  2-3  4 or more | 5.2%  4.3%  6.7%  4.9% | (ref)  0.83 (0.41-1.65)  1.27 (0.69-2.35)  0.93 (0.24-3.52) | (ref)  0.85 (0.43-1.70)  1.37 (0.75-2.52)  1.13 (0.30-4.26) | 11.2%  14.5%  15.6%  18.2% | (ref)  1.29 (0.82-2.03)  1.39 (0.90-2.16)  1.62 (0.82-3.21) | (ref)  1.33 (0.85-2.06)  1.47 (0.96-2.25)  1.87 (0.95-3.67) |
| **Number of medications****  0  1-2  3-4  5 or more | 3.1%  5.6%  6.5%  9.0% | (ref)  1.82 (0.87-3.82)  2.12 (0.97-4.62)  2.94 (1.02-8.50) | (ref)  1.87 (0.90-3.87)  2.47 (1.15-5.33)  3.39 (1.20-9.58) | 12.4%  13.8%  16.9%  14.1% | (ref)  1.11 (0.70-1.75)  1.37 (0.84-2.22)  1.14 (0.55-2.36) | (ref)  1.12 (0.71-1.77)  1.51 (0.93-2.47)  1.25 (0.60-2.58) |
| **Activities of daily living**  No difficulty  Difficulty | 5.5%  4.7% | (ref)  0.85 (0.34-2.16) | (ref)  1.32 (0.51-3.38) | 14.5%  13.5% | (ref)  0.93 (0.54-1.60) | (ref)  1.31 (0.76-2.24) |
| **Instrumental activities of daily living**  No difficulty  Difficulty | 6.0%  4.9% | (ref)  0.83 (0.50-1.39) | (ref)  1.14 (0.66-1.95) | 16.3%  12.4% | (ref)  0.76 (0.55-1.05) | (ref)  0.97 (0.68-1.38) |
| **Functional limitations**  No difficulty  Difficulty | 5.6%  5.2% | (ref)  0.93 (0.53-1.64) | (ref)  1.15 (0.65-2.02) | 15.3%  12.4% | (ref)  0.81 (0.57-1.16) | (ref)  0.95 (0.66-1.37) |
| **Smoking status**  Never smoker  Former smoker  Current smoker | 4.2%  9.6%  2.5% | (ref)  2.25 (1.35-3.77)  0.59 (0.17-2.07) | (ref)  2.00 (1.20-3.33)  0.56 (0.16-1.94) | 13.2%  20.2%  5.5% | (ref)  1.53 (1.10-2.13)  0.42 (0.19-0.91) | (ref)  1.39 (0.99-1.95)  0.40 (0.18-0.85) |
| **Body mass index**  Not obese  Obese | 5.3%  6.7% | (ref)  1.28 (0.61-2.68) | (ref)  1.13 (0.55-2.32) | 14.9%  11.3% | (ref)  0.76 (0.44-1.31) | (ref)  0.69 (0.41-1.17) |

Abbreviations: PR, prevalence ratio; aPR, adjusted prevalence ratio.

* based on 9 different morbidities: asthma, pulmonary disorders (bronchitis, COPD, and emphysema), hypertension, elevated cholesterol, diabetes, myocardial infarction, stroke, cancer, and depression.

** based on 10 different medication types: hypertension medication, heart medication, insomnia medication, pain medication, calming medication, attention medication, cholesterol medication, depression medication, diabetes medication, and osteoporosis medication.

# Appendix Table 15 – Proportions and prevalence ratios of breast and cervical cancer screening in the past 12 months in categories of health status indicators and age group among older women above 75 years of age (unweighted)

|  | **Mammography (women; n = 1,157)** | | | **Cervical smear (women; n = 1,157)** | | |
| --- | --- | --- | --- | --- | --- | --- |
| **Screening according to health indicators** | **Proportions** | **PR (unadjusted)** | **aPR (adjusted for age and sex)** | **Proportions** | **PR (unadjusted)** | **aPR (adjusted for age and sex)** |
| **Age group**  76-80  81-85  86+ | 54 (8.6%)  13 (3.7%)  4 (2.3%) | (ref)  0.43 (0.24-0.78)  0.26 (0.10-0.72) | -  -  - | 127 (20.2%)  44 (12.6%)  8 (4.5%) | (ref)  0.62 (0.45-0.86)  0.22 (0.11-0.45) | -  -  - |
| **Self-rated health**  Good or very good  Average  Bad or very bad | 51 (6.3%)  17 (5.7%)  3 (5.8%) | (ref)  0.90 (0.53-1.54)  0.91 (0.29-2.83) | (ref)  1.04 (0.64-1.77)  1.00 (0.34-2.95) | 133 (16.5%)  33 (11.1%)  13 (25.0%) | (ref)  0.67 (0.47-0.96)  1.52 (0.92-2.49) | (ref)  0.74 (0.52-1.06)  1.62 (1.00-2.63) |
| **Chronic condition or long-term health issue**  No  Yes | 30 (5.5%)  41 (6.7%) | (ref)  1.20 (0.76-1.89) | (ref)  1.27 (0.80-1.99) | 86 (15.9%)  93 (15.1%) | (ref)  0.95 (0.73-1.24) | (ref)  0.99 (0.76-1.29) |
| **Number of morbidities***  0  1  2-3  4 or more | 18 (6.4%)  19 (5.0%)  31 (7.4%)  3 (4.3%) | (ref)  0.78 (0.42-1.46)  1.16 (0.66-2.03)  0.67 (0.20-2.22) | (ref)  0.83 (0.45-1.55)  1.28 (0.74-2.25)  0.78 (0.24-2.55) | 36 (12.7%)  61 (15.9%)  71 (16.9%)  11 (15.7%) | (ref)  1.25 (0.85-1.84)  1.33 (0.91-1.92)  1.24 (0.66-2.30) | (ref)  1.31 (0.90-1.91)  1.43 (1.00-2.06)  1.37 (0.74-2.54) |
| **Number of medications****  0  1-2  3-4  5 or more | 11 (4.5%)  33 (6.2%)  22 (6.9%)  5 (7.8%) | (ref)  1.39 (0.71-2.70)  1.54 (0.76-3.12)  1.74 (0.63-4.83) | (ref)  1.44 (0.75-2.78)  1.75 (0.87-3.51)  1.99 (0.72-5.48) | 31 (12.7%)  85 (16.0%)  53 (16.7%)  10 (15.6%) | (ref)  1.27 (0.86-1.86)  1.32 (0.87-1.99)  1.23 (0.64-2.38) | (ref)  1.30 (0.89-1.90)  1.44 (0.96-2.16)  1.35 (0.71-2.59) |
| **Activities of daily living**  No difficulty  Difficulty | 66 (6.3%)  5 (4.9%) | (ref)  0.78 (0.32-1.88) | (ref)  1.20 (0.49-2.94) | 163 (15.5%)  16 (15.5%) | (ref)  1.00 (0.63-1.61) | (ref)  1.42 (0.89-2.28) |
| **Instrumental activities of daily living**  No difficulty  Difficulty | 41 (6.7%)  30 (5.5%) | (ref)  0.83 (0.53-1.32) | (ref)  1.08 (0.67-1.72) | 102 (16.6%)  77 (14.2%) | (ref)  0.86 (0.65-1.13) | (ref)  1.05 (0.79-1.39) |
| **Functional limitations**  No difficulty  Difficulty | 53 (6.5%)  18 (5.2%) | (ref)  0.81 (0.48-1.36) | (ref)  0.96 (0.57-1.62) | 134 (16.5%)  45 (13.1%) | (ref)  0.80 (0.58-1.09) | (ref)  0.92 (0.67-1.26) |
| **Smoking status**  Never smoker  Former smoker  Current smoker | 37 (4.8%)  31 (10.6%)  3 (3.1%) | (ref)  2.20 (1.39-3.48)  0.63 (0.20-2.02) | (ref)  2.02 (1.29-3.18)  0.60 (0.19-1.89) | 108 (14.1%)  64 (21.9%)  7 (7.1%) | (ref)  1.56 (1.18-2.06)  0.51 (0.24-1.06) | (ref)  1.46 (1.11-1.92)  0.48 (0.23-0.99) |
| **Body mass index**  Not obese  Obese | 62 (6.2%)  9 (5.9%) | (ref)  0.96 (0.49-1.89) | (ref)  0.91 (0.47-1.78) | 162 (16.1%)  17 (11.2%) | (ref)  0.69 (0.43-1.11) | (ref)  0.67 (0.42-1.06) |

Abbreviations: PR, prevalence ratio; aPR, adjusted prevalence ratio.

* based on 9 different morbidities: asthma, pulmonary disorders (bronchitis, COPD, and emphysema), hypertension, elevated cholesterol, diabetes, myocardial infarction, stroke, cancer, and depression.

** based on 10 different medication types: hypertension medication, heart medication, insomnia medication, pain medication, calming medication, attention medication, cholesterol medication, depression medication, diabetes medication, and osteoporosis medication.

# Appendix Table 16 – Proportions and prevalence ratios of prostate cancer screening in the past 12 months in categories of health status indicators and age group among older men above 75 years of age (weighted)

|  | **PSA or rectal exam (men; n=951)** | | |
| --- | --- | --- | --- |
| **Screening according to health indicators** | **Proportions** | **PR (unadjusted)** | **aPR (adjusted for age and sex)** |
| **Age group**  76-80  81-85  86+ | 23.6%  19.4%  10.7% | (ref)  0.82 (0.60-1.13)  0.45 (0.25-0.81) | -  -  - |
| **Self-rated health**  Good or very good  Average  Bad or very bad | 18.2%  25.9%  21.1% | (ref)  1.42 (1.02-1.99)  1.16 (0.54-2.46) | (ref)  1.37 (0.97-1.93)  1.19 (0.58-2.43) |
| **Chronic condition or long-term health issue**  No  Yes | 17.6%  21.9% | (ref)  1.25 (0.93-1.67) | (ref)  1.25 (0.93-1.67) |
| **Number of morbidities***  0  1  2-3  4 or more | 15.7%  18.6%  21.6%  23.5% | (ref)  1.18 (0.72-1.93)  1.37 (0.88-2.14)  1.49 (0.85-2.65) | (ref)  1.24 (0.76-2.01)  1.36 (0.87-2.11)  1.48 (0.84-2.59) |
| **Number of medications****  0  1-2  3-4  5 or more | 14.7%  18.5%  26.0%  16.5% | (ref)  1.26 (0.80-1.99)  1.77 (1.12-2.81)  1.12 (0.48-2.64) | (ref)  1.29 (0.82-2.02)  1.79 (1.13-2.82)  1.17 (0.50-2.71) |
| **Activities of daily living**  No difficulty  Difficulty | 20.3%  14.5% | (ref)  0.71 (0.36-1.40) | (ref)  0.78 (0.40-1.55) |
| **Instrumental activities of daily living**  No difficulty  Difficulty | 21.1%  17.4% | (ref)  0.82 (0.59-1.14) | (ref)  0.89 (0.64-1.23) |
| **Functional limitations**  No difficulty  Difficulty | 19.9%  19.9% | (ref)  1.00 (0.72-1.39) | (ref)  1.08 (0.78-1.49) |
| **Smoking status**  Never smoker  Former smoker  Current smoker | 19.3%  21.3%  14.9% | (ref)  1.10 (0.81-1.50)  0.77 (0.44-1.34) | (ref)  1.09 (0.80-1.48)  0.70 (0.40-1.22) |
| **Body mass index**  Not obese  Obese | 19.2%  24.8% | (ref)  1.29 (0.89-1.89) | (ref)  1.20 (0.82-1.74) |

Abbreviations: Prostate specific antigen test, PSA; PR, prevalence ratio; aPR, adjusted prevalence ratio.

* based on 9 different morbidities: asthma, pulmonary disorders (bronchitis, COPD, and emphysema), hypertension, elevated cholesterol, diabetes, myocardial infarction, stroke, cancer, and depression.

** based on 10 different medication types: hypertension medication, heart medication, insomnia medication, pain medication, calming medication, attention medication, cholesterol medication, depression medication, diabetes medication, and osteoporosis medication.

# Appendix Table 17 – Proportions and prevalence ratios of prostate cancer screening in the past 12 months in categories of health status indicators and age group among older men above 75 years of age (unweighted)

|  | **PSA or rectal exam (men; n=951)** | | |
| --- | --- | --- | --- |
| **Screening according to health indicators** | **Proportions** | **PR (unadjusted)** | **aPR (adjusted for age and sex)** |
| **Age group**  76-80  81-85  86+ | 131 (24.4%)  62 (21.5%)  16 (12.6%) | (ref)  0.88 (0.68-1.15)  0.52 (0.32-0.83) | -  -  - |
| **Self-rated health**  Good or very good  Average  Bad or very bad | 154 (21.3%)  47 (24.4%)  8 (22.9%) | (ref)  1.14 (0.86-1.52)  1.07 (0.57-2.00) | (ref)  1.13 (0.85-1.51)  1.09 (0.58-2.05) |
| **Chronic condition or long-term health issue**  No  Yes | 89 (20.0%)  120 (23.7%) | (ref)  1.18 (0.93-1.51) | (ref)  1.18 (0.92-1.50) |
| **Number of morbidities***  0  1  2-3  4 or more | 28 (14.9%)  53 (21.0%)  103 (24.5%)  25 (27.8%) | (ref)  1.41 (0.93-2.14)  1.64 (1.12-2.40)  1.87 (1.16-3.01) | (ref)  1.42 (0.93-2.15)  1.61 (1.10-2.36)  1.83 (1.13-2.95) |
| **Number of medications****  0  1-2  3-4  5 or more | 27 (13.6%)  94 (21.7%)  80 (28.1%)  8 (22.9%) | (ref)  1.59 (1.07-2.36)  2.06 (1.38-3.06)  1.68 (0.83-3.38) | (ref)  1.62 (1.09-2.40)  2.08 (1.40-3.08)  1.68 (0.83-3.41) |
| **Activities of daily living**  No difficulty  Difficulty | 197 (22.3%)  12 (18.2%) | (ref)  0.82 (0.48-1.38) | (ref)  0.87 (0.51-1.47) |
| **Instrumental activities of daily living**  No difficulty  Difficulty | 150 (23.0%)  59 (19.7%) | (ref)  0.85 (0.65-1.12) | (ref)  0.90 (0.69-1.18) |
| **Functional limitations**  No difficulty  Difficulty | 152 (22.3%)  57 (21.1%) | (ref)  0.95 (0.72-1.24) | (ref)  0.99 (0.76-1.30) |
| **Smoking status**  Never smoker  Former smoker  Current smoker | 77 (19.8%)  112 (24.2%)  20 (20.0%) | (ref)  1.22 (0.94-1.58)  1.01 (0.65-1.56) | (ref)  1.19 (0.92-1.54)  0.96 (0.62-1.48) |
| **Body mass index**  Not obese  Obese | 173 (21.0%)  36 (28.1%) | (ref)  1.34 (0.98-1.82) | (ref)  1.28 (0.94-1.74) |

Abbreviations: Prostate specific antigen test, PSA; PR, prevalence ratio; aPR, adjusted prevalence ratio.

* based on 9 different morbidities: asthma, pulmonary disorders (bronchitis, COPD, and emphysema), hypertension, elevated cholesterol, diabetes, myocardial infarction, stroke, cancer, and depression.

** based on 10 different medication types: hypertension medication, heart medication, insomnia medication, pain medication, calming medication, attention medication, cholesterol medication, depression medication, diabetes medication, and osteoporosis medication.

# Appendix Table 18 - Sensitivity analysis: Proportions and prevalence ratios of any cancer screening in the past 12 months in categories of number of morbidities among all older adults above 75 years of age (weighted)

|  | **All older adults (n = 2,108)** | | |
| --- | --- | --- | --- |
| **Screening according to health indicators** | **Proportions** | **PR (unadjusted)** | **aPR (adjusted for age and sex)** |
| **Number of morbidities* (w/o hypertension)**  0  1  2-3  4 or more | 18.7%  26.5%  28.5%  21.1% | (ref)  1.41 (1.13-1.76)  1.52 (1.22-1.90)  1.13 (0.62-2.06) | (ref)  1.38 (1.11-1.72)  1.44 (1.16-1.79)  1.12 (0.61-2.06) |
| **Number of morbidities* (w/o elevated cholesterol)**  0  1  2-3  4 or more | 21.3%  22.3%  28.6%  21.0% | (ref)  1.05 (0.83-1.33)  1.34 (1.07-1.69)  0.99 (0.56-1.73) | (ref)  1.06 (0.84-1.33)  1.33 (1.06-1.67)  1.01 (0.59-1.76) |
| **Number of morbidities* (w/o hypertension and elevated cholesterol)**  0  1  2+ | 21.4%  26.0%  28.2% | (ref)  1.21 (1.00-1.47)  1.32 (1.03-1.68) | (ref)  1.17 (0.96-1.42)  1.32 (1.04-1.67) |

* based on 9 different morbidities: asthma, pulmonary disorders (bronchitis, COPD, and emphysema), hypertension, elevated cholesterol, diabetes, myocardial infarction, stroke, cancer, and depression.

# Appendix Table 19 – Sensitivity analysis: Proportions and prevalence ratios of any cancer screening in the past 12 months according to number of morbidities among older men and women above 75 years of age (weighted)

|  | **Men (n=951)** | | | **Women (n=1,157)** | | |
| --- | --- | --- | --- | --- | --- | --- |
| **Screening according to health indicators** | **Proportions** | **PR (unadjusted)** | **aPR (adjusted for age)** | **Proportions** | **PR (unadjusted)** | **aPR (adjusted for age)** |
| **Number of morbidities* (w/o hypertension)**  0  1  2-3  4 or more | 22.9%  27.9%  31.9%  29.1% | (ref)  1.22 (0.89-1.67)  1.40 (1.03-1.89)  1.27 (0.66-2.45) | (ref)  1.24 (0.91-1.69)  1.35 (1.00-1.81)  1.33 (0.69-2.56) | 16.0%  25.1%  24.8%  8.2% | (ref)  1.57 (1.16-2.14)  1.55 (1.12-2.16)  0.51 (0.08-3.32) | (ref)  1.52 (1.12-2.06)  1.56 (1.13-2.15)  0.52 (0.08-3.52) |
| **Number of morbidities* (w/o elevated cholesterol)**  0  1  2-3  4 or more | 23.8%  26.9%  30.3%  31.9% | (ref)  1.13 (0.81-1.59)  1.27 (0.91-1.78)  1.34 (0.73-2.47) | (ref)  1.13 (0.81-1.58)  1.24 (0.89-1.72)  1.37 (0.76-2.49) | 19.5%  18.5%  26.9%  6.0% | (ref)  0.95 (0.68-1.32)  1.38 (1.00-1.91)  0.31 (0.05-2.05) | (ref)  0.99 (0.72-1.37)  1.51 (1.11-2.06)  0.36 (0.05-2.45) |
| **Number of morbidities* (w/o hypertension and elevated cholesterol)**  0  1  2+ | 26.3%  26.7%  32.9% | (ref)  1.01 (0.77-1.33)  1.25 (0.92-1.71) | (ref)  0.98 (0.75-1.29)  1.24 (0.91-1.68) | 17.7%  25.3%  23.5% | (ref)  1.43 (1.07-1.89)  1.33 (0.91-1.94) | (ref)  1.42 (1.07-1.87)  1.43 (0.98-2.10) |

Abbreviations : PR, prevalence ratio ; aPR, adjusted prevalence ratio.

* based on 9 different morbidities: asthma, pulmonary disorders (bronchitis, COPD, and emphysema), hypertension, elevated cholesterol, diabetes, myocardial infarction, stroke, cancer, and depression.

# Appendix Table 20 - Sensitivity analysis: Proportions and prevalence ratios of any cancer screening in the past 12 months in categories of number of morbidities among all older adults above 75 years of age (unweighted)

|  | **All older adults (n = 2,108)** | | |
| --- | --- | --- | --- |
| **Screening according to health indicators** | **Proportions** | **PR (unadjusted)** | **aPR (adjusted for age and sex)** |
| **Number of morbidities* (w/o hypertension)**  0  1  2-3  4 or more | 162 (20.0%)  193 (27.3%)  172 (31.7%)  12 (24.5%) | (ref)  1.36 (1.13-1.63)  1.58 (1.31-1.90)  1.22 (0.74-2.04) | (ref)  1.33 (1.11-1.59)  1.48 (1.23-1.78)  1.17 (0.71-1.94) |
| **Number of morbidities* (w/o elevated cholesterol)**  0  1  2-3  4 or more | 121 (21.8%)  205 (24.4%)  199 (30.8%)  14 (21.9%) | (ref)  1.12 (0.92-1.36)  1.41 (1.16-1.72)  1.01 (0.62-1.64) | (ref)  1.13 (0.93-1.37)  1.39 (1.14-1.69)  1.01 (0.63-1.63) |
| **Number of morbidities* (w/o hypertension and elevated cholesterol)**  0  1  2+ | 252 (22.9%)  192 (26.8%)  95 (32.8%) | (ref)  1.17 (1.00-1.38)  1.43 (1.18-1.75) | (ref)  1.13 (0.96-1.32)  1.41 (1.16-1.71) |

* based on 9 different morbidities: asthma, pulmonary disorders (bronchitis, COPD, and emphysema), hypertension, elevated cholesterol, diabetes, myocardial infarction, stroke, cancer, and depression.

# Appendix Table 21 – Sensitivity analysis: Proportions and prevalence ratios of any cancer screening in the past 12 months according to number of morbidities among older men and women above 75 years of age (unweighted prevalence ratios)

|  | **Men (n=951)** | | | **Women (n=1,157)** | | |
| --- | --- | --- | --- | --- | --- | --- |
| **Screening according to health indicators** | **Proportions** | **PR (unadjusted)** | **aPR (adjusted for age)** | **Proportions** | **PR (unadjusted)** | **aPR (adjusted for age)** |
| **Number of morbidities* (w/o hypertension)**  0  1  2-3  4 or more | 73 (23.4%)  99 (31.3%)  104 (35.3%)  11 (39.3%) | (ref)  1.34 (1.03-1.73)  1.51 (1.17-1.94)  1.68 (1.02-2.77) | (ref)  1.33 (1.03-1.72)  1.47 (1.14-1.88)  1.69 (1.02-2.82) | 89 (17.9%)  94 (24.0%)  68 (27.4%)  1 (4.8%) | (ref)  1.34 (1.03-1.73)  1.53 (1.16-2.01)  0.27 (0.04-1.81) | (ref)  1.33 (1.03-1.71)  1.54 (1.18-2.03)  0.27 (0.04-1.88) |
| **Number of morbidities* (w/o elevated cholesterol)**  0  1  2-3  4 or more | 51 (22.5%)  112 (31.1%)  111 (33.5%)  13 (39.4%) | (ref)  1.38 (1.04-1.84)  1.49 (1.12-1.99)  1.75 (1.08-2.85) | (ref)  1.37 (1.03-1.82)  1.46 (1.10-1.94)  1.76 (1.08-2.86) | 70 (21.3%)  93 (19.3%)  88 (27.8%)  1 (3.2%) | (ref)  0.91 (0.69-1.20)  1.31 (1.00-1.72)  0.15 (0.02-1.05) | (ref)  0.97 (0.74-1.28)  1.42 (1.09-1.86)  0.17 (0.02-1.20) |
| **Number of morbidities* (w/o hypertension and elevated cholesterol)**  0  1  2+ | 128 (28.3%)  100 (28.8%)  59 (39.1%) | (ref)   - 1. (0.82-1.27)   1.38 (1.08-1.77) | (ref)  1.00 (0.80-1.24)  1.38 (1.08-1.76) | 124 (19.1%)  92 (24.9%)  36 (25.9%) | (ref)  1.30 (1.03-1.65)  1.36 (0.98-1.87) | (ref)  1.31 (1.04-1.65)  1.43 (1.04-1.96) |

Abbreviations: PR, prevalence ratio; aPR, adjusted prevalence ratio.

* based on 9 different health morbidities: asthma, pulmonary disorders (bronchitis, COPD, and emphysema), hypertension, elevated cholesterol, diabetes, myocardial infarction, stroke, cancer, and depression.

# Appendix Table 22 – Proportions of study participants in body mass index categories with alternative categorizations (weighted)

| **Body weight categories** | **All**  **(n=2,108)** | **Men**  **(n=951)** | **Women**  **(n=1,157)** |
| --- | --- | --- | --- |
| **Body mass index**  Underweight (BMI < 18.5)  Normal weight (18.5 <= BMI < 25)  Overweight (25 <= BMI < 30)  Obese (30 <= BMI) | 3.2%  48.4%  35.2%  13.2% | 0.6%  43.4%  43.3%  12.7% | 52.6%  5.5%  28.2%  13.7% |
| **Body mass index**  Underweight or normal weight (BMI < 25)  Overweight or obese (25 <= 25) | 51.6%  48.4% | 44.0%  56.0% | 58.1%  41.9% |

# Appendix Table 23 – Proportions of any cancer screening in the past 12 months in granular body mass index categories among older adults above 75 years of age (weighted)

| **Body weight categories** | **Any cancer screening**  **(all older adults; n=2,108)** | **Any cancer screening**  **(men; n=951)** | **Any cancer screening**  **(women; n=1,157)** |
| --- | --- | --- | --- |
| **Body mass index**  Underweight (BMI < 18.5)  Normal weight (18.5 <= BMI < 25)  Overweight (25 <= BMI < 30)  Obese (30 <= BMI) | 13.6%  22.2%  26.3%  27.2% | 18.2%  24.6%  28.3%  35.4% | 13.2%  20.5%  23.6%  20.7% |

# Appendix Table 24 – Proportions and prevalence ratios of any cancer screening in the past 12 months in body mass index categories with alternative categorization of body mass index among older adults above 75 years of age (weighted)

|  | **All older adults (n = 2,108)** | | |
| --- | --- | --- | --- |
| **Screening according to health indicators** | **Proportions** | **PR (unadjusted)** | **aPR (adjusted for age and sex)** |
| **Body mass index**  Underweight or normal weight  Overweight or obese | 21.7%  26.5% | 0.82 (0.68-0.97)  (ref) | 0.90 (0.75-1.07)  (ref) |

# Appendix Table 25 – Proportions and prevalence ratios of any cancer screening in the past 12 months in body mass index categories with alternative categorization of body mass index among older men and women above 75 years of age (weighted)

|  | **Men (n=951)** | | | **Women (n=1,157)** | | |
| --- | --- | --- | --- | --- | --- | --- |
| **Screening according to health indicators** | **Proportions** | **PR (unadjusted)** | **aPR (adjusted for age and sex)** | **Proportions** | **PR (unadjusted)** | **aPR (adjusted for age and sex)** |
| **Body mass index**  Underweight or normal weight  Overweight or obese | 24.5%  29.9% | 0.82 (0.64-1.05)  (ref) | 0.87 (0.69-1.11)  (ref) | 19.8%  22.6% | 0.87 (0.67-1.14)  (ref) | 0.91 (0.70-1.18)  (ref) |

# Appendix Table 26 – Proportions of any cancer screening in the past 12 months in granular body mass index categories among older adults above 75 years of age (unweighted)

| **Body weight categories** | **Any cancer screening**  **(all older adults; n=2,108)** | **Any cancer screening**  **(men; n=951)** | **Any cancer screening**  **(women; n=1,157)** |
| --- | --- | --- | --- |
| **Body mass index**  Underweight (BMI < 18.5)  Normal weight (18.5 <= BMI < 25)  Overweight (25 <= BMI < 30)  Obese (30 <= BMI) | 10 (14.9%)  248 (23.9%)  206 (28.5%)  75 (26.8%) | 2 (33.3%)  108 (26.3%)  128 (31.5%)  49 (38.3%) | 8 (13.1%)  140 (22.4%)  78 (24.5%)  26 (17.1%) |

# Appendix Table 27 – Proportions and prevalence ratios of any cancer screening in the past 12 months in body mass index categories with alternative categorization of body mass index among older adults above 75 years of age (unweighted)

|  | **All older adults (n = 2,108)** | | |
| --- | --- | --- | --- |
| **Screening according to health indicators** | **Proportions** | **PR (unadjusted)** | **aPR (adjusted for age and sex)** |
| **Body mass index**  Underweight or normal weight  Overweight or obese | 258 (23.4%)  281 (28.0%) | 0.83 (0.72-0.97)  (ref) | 0.92 (0.80-1.07)  (ref) |

# Appendix Table 28 – Proportions and prevalence ratios of any cancer screening in the past 12 months in body mass index categories with alternative categorization of body mass index among older men and women above 75 years of age (unweighted)

|  | **Men (n=951)** | | | **Women (n=1,157)** | | |
| --- | --- | --- | --- | --- | --- | --- |
| **Screening according to health indicators** | **Proportions** | **PR (unadjusted)** | **aPR (adjusted for age and sex)** | **Proportions** | **PR (unadjusted)** | **aPR (adjusted for age and sex)** |
| **Body mass index**  Underweight or normal weight  Overweight or obese | 110 (26.4%)  177 (33.1%) | 0.80 (0.65-0.97)  (ref) | 0.84 (0.68-1.02)  (ref) | 148 (21.5%)  104 (22.1%) | 0.97 (0.78-1.22)  (ref) | 1.01 (0.81-1.26)  (ref) |

# Appendix Table 29 – Proportions of colorectal cancer screening in the past 12 months in granular body mass index categories among older adults above 75 years of age (weighted)

| **Body weight categories** | **Any colorectal**  **(all older adults; n=2,108)** | **FOBT**  **(all older adults; n=2,108)** | **Colonoscopy**  **(all older adults; n=2,108)** |
| --- | --- | --- | --- |
| **Body mass index**  Underweight (BMI < 18.5)  Normal weight (18.5 <= BMI < 25)  Overweight (25 <= BMI < 30)  Obese (30 <= BMI) | 2.1%  7.6%  11.9%  11.0% | 0.0%  4.0%  6.9%  7.7% | 2.1%  4.4%  6.3%  7.7% |

Abbreviations: Fecal occult blood test, FOBT.

# Appendix Table 30 – Proportions and prevalence ratios of any colorectal cancer screening in the past 12 months in body mass index categories with alternative categorization of body mass index among older adults above 75 years of age (weighted)

|  | **Any colorectal (all older adults; n = 2,108)** | | |
| --- | --- | --- | --- |
| **Screening according to health indicators** | **Proportions** | **PR (unadjusted)** | **aPR (adjusted for age and sex)** |
| **Body mass index**  Underweight or normal weight  Overweight or obese | 11.2%  13.4% | 0.62 (0.45-0.85)  (ref) | 0.71 (0.51-0.98)  (ref) |

# Appendix Table 31 – Proportions and prevalence ratios of FOBT and colonoscopy screening in the past 12 months in body mass index categories with alternative categorization of body mass index among older adults above 75 years of age (weighted)

|  | **FOBT (all older adults; n = 2,108)** | | | **Colonoscopy (all older adults; n = 2,108)** | | |
| --- | --- | --- | --- | --- | --- | --- |
| **Screening according to health indicators** | **Proportions** | **PR (unadjusted)** | **aPR (adjusted for age and sex)** | **Proportions** | **PR (unadjusted)** | **aPR (adjusted for age and sex)** |
| **Body mass index**  Underweight or normal weight  Overweight or obese | 3.7%  7.1% | 0.52 (0.33-0.81)  (ref) | 0.59 (0.37-0.93)  (ref) | 4.3%  6.7% | 0.64 (0.41-0.98)  (ref) | (0.72-0.46)  (ref) |

Abbreviations: Fecal occult blood test, FOBT.

# Appendix Table 32 – Proportions of colorectal cancer screening in the past 12 months in granular body mass index categories among older adults above 75 years of age (unweighted)

| **Body weight categories** | **Any colorectal**  **(all older adults; n=2,108)** | **FOBT**  **(all older adults; n=2,108)** | **Colonoscopy**  **(all older adults; n=2,108)** |
| --- | --- | --- | --- |
| **Body mass index**  Underweight (BMI < 18.5)  Normal weight (18.5 <= BMI < 25)  Overweight (25 <= BMI < 30)  Obese (30 <= BMI) | 2 (3.0%)  78 (7.5%)  85 (11.7%)  29 (10.4%) | 0 (0.0%)  41 (4.0%)  50 (6.9%)  18 (6.4%) | 2 (3.0%)  46 (4.4%)  47 (6.5%)  18 (6.4%) |

Abbreviations: Fecal occult blood test, FOBT.

# Appendix Table 33 – Proportions and prevalence ratios of any colorectal cancer screening in the past 12 months in body mass index categories with alternative categorization of body mass index among older adults above 75 years of age (unweighted)

|  | **Any colorectal (all older adults; n = 2,108)** | | |
| --- | --- | --- | --- |
| **Screening according to health indicators** | **Proportions** | **PR (unadjusted)** | **aPR (adjusted for age and sex)** |
| **Body mass index**  Underweight or normal weight  Overweight or obese | 80 (7.2%)  114 (11.4%) | 0.64 (0.49-0.84)  (ref) | 0.75 (0.57-0.99)  (ref) |

# Appendix Table 34 – Proportions and prevalence ratios of FOBT and colonoscopy screening in the past 12 months in body mass index categories with alternative categorization of body mass index among older adults above 75 years of age (unweighted)

|  | **FOBT (all older adults; n = 2,108)** | | | **Colonoscopy (all older adults; n = 2,108)** | | |
| --- | --- | --- | --- | --- | --- | --- |
| **Screening according to health indicators** | **Proportions** | **PR (unadjusted)** | **aPR (adjusted for age and sex)** | **Proportions** | **PR (unadjusted)** | **aPR (adjusted for age and sex)** |
| **Body mass index**  Underweight or normal weight  Overweight or obese | 41 (3.7%)  68 (6.8%) | 0.55 (0.38-0.80)  (ref) | 0.64 (0.44-0.94)  (ref) | 48 (4.3%)  65 (6.5%) | 0.67 (0.47-1.16)  (ref) | 0.79 (0.55-1.15)  (ref) |

Abbreviations: Fecal occult blood test, FOBT.

# Appendix Table 35 – Proportions of breast, cervical, and prostate cancer screening in the past 12 months in granular body mass index categories among older adults above 75 years of age (weighted)

| **Body weight categories** | **Mammography**  **(women; n=1,157)** | **Cervical smear**  **(women; n=1,157)** | **PSA or rectal exam**  **(men; n=951)** |
| --- | --- | --- | --- |
| **Body mass index**  Underweight (BMI < 18.5)  Normal weight (18.5 <= BMI < 25)  Overweight (25 <= BMI < 30)  Obese (30 <= BMI) | 0.9%  5.1%  6.4%  6.7% | 10.0%  15.3%  15.1%  11.3% | 18.2%  17.5%  20.8%  24.8% |

Abbreviations: Prostate specific antigen test, PSA.

# Appendix Table 36 – Proportions and prevalence ratios of breast and cervical cancer screening in the past 12 months in body mass index categories with alternative categorization of body mass index among older women above 75 years of age (weighted)

|  | **Mammography (women; n = 1,157)** | | | **Cervical smear (women; n = 1,157)** | | |
| --- | --- | --- | --- | --- | --- | --- |
| **Screening according to health indicators** | **Proportions** | **PR (unadjusted)** | **aPR (adjusted for age and sex)** | **Proportions** | **PR (unadjusted)** | **aPR (adjusted for age and sex)** |
| **Body mass index**  Underweight or normal weight  Overweight or obese | 4.7%  6.5% | 0.73 (0.44-1.20)  (ref) | 0.77 (0.47-1.27)  (ref) | 14.8%  13.8% | 1.07 (0.77-1.49)  (ref) | 1.12 (0.81-1.55)  (ref) |

# Appendix Table 37 – Proportions and prevalence ratios of prostate cancer screening in the past 12 months in body mass index categories with alternative categorization of body mass index among older men above 75 years of age (weighted)

|  | **PSA or rectal exam (men; n=951)** | | |
| --- | --- | --- | --- |
| **Screening according to health indicators** | **Proportions** | **PR (unadjusted)** | **aPR (adjusted for age and sex)** |
| **Body mass index**  Underweight or normal weight  Overweight or obese | 17.5%  21.7% | 0.81 (0.60-1.09)  (ref) | 0.87 (0.65-1.16)  (ref) |

Abbreviations: Prostate specific antigen test, PSA.

# Appendix Table 38 – Proportions of breast, cervical, and prostate cancer screening in the past 12 months in granular body mass index categories among older adults above 75 years of age (unweighted)

| **Body weight categories** | **Mammography**  **(women; n=1,157)** | **Cervical smear**  **(women; n=1,157)** | **PSA or rectal exam**  **(men; n=951)** |
| --- | --- | --- | --- |
| **Body mass index**  Underweight (BMI < 18.5)  Normal weight (18.5 <= BMI < 25)  Overweight (25 <= BMI < 30)  Obese (30 <= BMI) | 1 (5.9%)  37 (1.6%)  24 (7.5%)  9 (5.9%) | 5 (8.2%)  105 (16.8%)  52 (16.4%)  17 (11.2%) | 2 (33.3%)  76 (18.5%)  95 (23.4%)  36 (28.1%) |

Abbreviations: Prostate specific antigen test, PSA.

# Appendix Table 39 – Proportions and prevalence ratios of breast and cervical cancer screening in the past 12 months in body mass index categories with alternative categorization of body mass index among older women above 75 years of age (unweighted)

|  | **Mammography (women; n = 1,157)** | | | **Cervical smear (women; n = 1,157)** | | |
| --- | --- | --- | --- | --- | --- | --- |
| **Screening according to health indicators** | **Proportions** | **PR (unadjusted)** | **aPR (adjusted for age and sex)** | **Proportions** | **PR (unadjusted)** | **aPR (adjusted for age and sex)** |
| **Body mass index**  Underweight or normal weight  Overweight or obese | 38 (5.5%)  33 (7.0%) | 0.79 (0.50-1.24)  (ref) | 0.83 (0.53-1.29)  (ref) | 110 (16.0%)  69 (14.7%) | 1.09 (0.83-1.44)  (ref) | 1.14 (0.86-1.49)  (ref) |

# Appendix Table 40 – Proportions and prevalence ratios of prostate cancer screening in the past 12 months in body mass index categories with alternative categorization of body mass index among older men above 75 years of age (unweighted)

|  | **PSA or rectal exam (men; n=951)** | | |
| --- | --- | --- | --- |
| **Screening according to health indicators** | **Proportions** | **PR (unadjusted)** | **aPR (adjusted for age and sex)** |
| **Body mass index**  Underweight or normal weight  Overweight or obese | 78 (18.7%)  131 (24.5%) | 0.76 (0.59-0.98)  (ref) | 0.80 (0.62-1.03)  (ref) |

Abbreviations: Prostate specific antigen test, PSA.

# Supplemental material references

Cho H, Wang Z, Yabroff KR, et al. Estimating life expectancy adjusted by self-rated health status in the United States: national health interview survey linked to the mortality. BMC Public Health. 2022;22(1):141. doi:10.1186/s12889-021-12332-0

Cruz M, Covinsky K, Widera EW, Stijacic-Cenzer I, Lee SJ. Predicting 10-Year Mortality for Older Adults. JAMA. 2013;309(9):874. doi:10.1001/jama.2013.1184

Gastens V, Chiolero A, Feller M, Bauer DC, Rodondi N, Del Giovane C. Development and internal validation of a new life expectancy estimator for multimorbid older adults. Diagn Progn Res. 2025;9(1):5. doi:10.1186/s41512-025-00185-9

Kobayashi LC, Jackson SE, Lee SJ, Wardle J, Steptoe A. The development and validation of an index to predict 10-year mortality risk in a longitudinal cohort of older English adults. Age Ageing. 2017;46(3):427-432. doi:10.1093/ageing/afw199

Yourman LC, Lee SJ, Schonberg MA, Widera EW, Smith AK. Prognostic indices for older adults: a systematic review. JAMA. 2012;307(2):182-192. doi:10.1001/jama.2011.1966
